# Transition-Metal-Catalyzed Transformations for the Synthesis of Marine Drugs

**DOI:** 10.3390/md22060253

**Published:** 2024-05-29

**Authors:** Lucía G. Parte, Sergio Fernández, Eva Sandonís, Javier Guerra, Enol López

**Affiliations:** 1 Department of Organic Chemistry, Science Faculty, University of Valladolid (UVa), Paseo de Belén 7, 47011 Valladolid, Spain; lucia.garcia.parte@uva.es (L.G.P.); eva.sandonis@estudiantes.uva.es (E.S.); 2 Department of Chemistry, School of Physical and Chemical Sciences, Queen Mary University of London (QMUL), Mile End Road, London E1 4NS, UK; s.f.gonzalez@qmul.ac.uk; 3 Department of Organic Chemistry, ITAP, School of Engineering (EII), University of Valladolid (UVa), Dr Mergelina, 47002 Valladolid, Spain

**Keywords:** transition metals, catalytic transformations, marine drugs, bioactive molecules

## Abstract

Transition metal catalysis has contributed to the discovery of novel methodologies and the preparation of natural products, as well as new chances to increase the chemical space in drug discovery programs. In the case of marine drugs, this strategy has been used to achieve selective, sustainable and efficient transformations, which cannot be obtained otherwise. In this perspective, we aim to showcase how a variety of transition metals have provided fruitful couplings in a wide variety of marine drug-like scaffolds over the past few years, by accelerating the production of these valuable molecules.

## 1. Introduction

The development of new chemical transformations that can provide organic chemists with appropriate tools to face synthetic challenges is always desirable by the scientific community. In this regard, catalysis has changed the way of making chemistry. It provides not only a unique green chemistry perspective in the construction of organic molecules by reducing cost and time through alternative reaction pathways but also efficient methodologies with increased yield and selectivities. With this in mind, a plethora of new catalytic transformations have been developed to achieve the preparation of organic scaffolds in both homogeneous and heterogeneous systems [1,2]. Transition metals and organocatalysts have governed these catalytic methodologies reported during the last decades due to the several advantages shown by these catalysts. In fact, various authors who reported these strategies have been recognized with the Nobel Prize in several years: 2001 (for stereoselective catalysis), 2005 (for olefin metathesis), 2010 (for palladium cross-coupling reactions) and 2021 (for asymmetric organocatalysis). 

On the one hand, organocatalysis accelerates organic reactions through the addition of stochiometric amounts of a small chiral organic compound. It has been highly considered for asymmetric transformations in the last 20 years because of the rapid advances in the field [3,4]. On the other hand, the ability of transition metal complexes to change easily between many oxidation states makes them very convenient for the development of new catalytic pathways. These reactions usually require low catalyst loadings (sometimes less than 1% or even traces), and these are very valuable to achieve challenging couplings and provide new opportunities in terms of (i) chemo- and regioselectivity, which can be tuned by substituting with other suitable transition metal or modifying the electronic and steric properties of the ligands; (ii) asymmetric catalysis, by using chiral transition metal complexes or additional ligands as chiral auxiliaries; and (iii) reactivity, by accessing new chemical space not accessible by alternative methodologies [5].

The potential of transition metal complexes to promote chemical reactions has been demonstrated in several types of very useful transformations such as C-H bond activation, cyclization reactions, cross-coupling reactions, metal carbene chemistry or in combination with novel synthetic tools, such as photo- and electrochemistry [6,7,8,9,10,11,12,13]. This chemical versatility makes transition metal complexes a valuable tool not only in the development of new catalytic methodologies but also in the preparation of natural products and pharmaceuticals with long multistep synthetic reactions, often implying a real challenge and a time-consuming process for an organic chemist. Transition metals have been used for the synthesis of libraries, discovery synthesis and the preparation of active pharmaceutical ingredients (APIs) on a large scale. Depending on the transition metal, high selectivities and functional group tolerances can be achieved in a plethora of chemical transformations. The most common have been carbon–carbon and carbon–heteroatom bond formations, oxidations, metathesis and asymmetric additions or hydrogenations. In this manner, preclinical and clinical candidates can efficiently be prepared using this approach [14,15,16]. At the end of the process, possible traces of the metal catalysts must be removed from the final products, usually through resin scavengers and filtration techniques until tolerated amounts to afford metal-free organic molecules.

In this context of elaborating organic scaffolds with pharmacological activities, marine drugs have been widely studied. They are commonly known as organic molecules that possess a high structural complexity with pharmacological properties that have been isolated from a diverse set of living organisms in the ocean, such as bacteria, mollusks, plants, invertebrates or sponges. These chemical entities have been screened against multiple cell lines, providing in some cases interesting biological activities and making them very convenient for further studies in the search for new therapies [17,18,19]. In many cases, they are sophisticated skeletons whose drug-like properties are caused by the biochemical or physiological evolution pathways that living organisms undergo to survive and reproduce in different environments over the years. However, due to the low bioavailability that can be obtained from marine sources, many efforts have been made to design suitable synthetic pathways for the construction of marine drugs and their analogs. Thus, both technological and chemical approaches have been developed to obtain these molecules synthetically [5,20,21].

The aim of this perspective is to showcase how transition-metal-catalyzed transformations can help in the complex synthesis of marine drugs. Although there is a myriad of methodologies that use transition metals to achieve these molecules, we have selected some representative examples, in which the final product has significant biological activity. For a better understanding of the work, this review is divided into different sections attending to the nature of each transition metal, in which a brief introduction of the most common metal-catalyzed processes is also disclosed.

## 2. Results

### 2.1. Cross-Coupling Reactions in the Synthesis of Marine Drugs

**Scheme 1:**
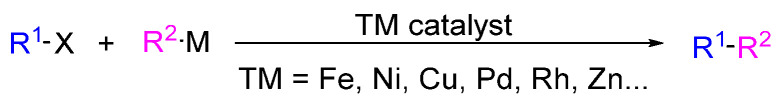
General scheme for cross-coupling reactions, where the fragments that are coupled in this reaction are indicated by the colors blue and pink.

Cross-coupling reactions are one of the most powerful and important methods for the creation of C-C bonds. These reactions occur when two reagents, both with activating groups, react together in the presence of a metal catalyst to form a new covalent bond, driven by the loss of the activating groups [22]. The metal catalyst is commonly a group 8–10 metal, and the reaction leads to the desired product, hopefully with high yields and selectivity. Initially, this methodology was very limited, and only Grignard reagents and organolithiums were used, while organic halides were restricted to alkyl halides. However, in the last few years, cross-coupling reactions have been extensively studied, and nowadays, a great variety of metal complexes as catalysts, organometallic reagents as nucleophiles and organic electrophiles (alkyl, aryl, alkenyl, alkynyl and allyl moieties bearing good leaving groups) can be employed (Scheme 1) [23].

The most widely used transition metals in cross-coupling reactions are those belonging to group 10, especially nickel and palladium. Of them, palladium is the most popular due to its higher activity, selectivity and reactivity. Furthermore, palladium complexes have a high tolerance for a wide range of functional groups on both organometallic and organic halides, and this allows for the synthesis of complex organic building blocks to be performed in fewer steps. These facts have boosted the development of a wide number of palladium catalysts with different ligands, allowing the fine-tuning of reactions [24,25]. Also, the combination of palladium catalysts with less reactive organometallic reagents has been demonstrated to be highly attractive in cross-coupling reactions [23].

**Scheme 2:**
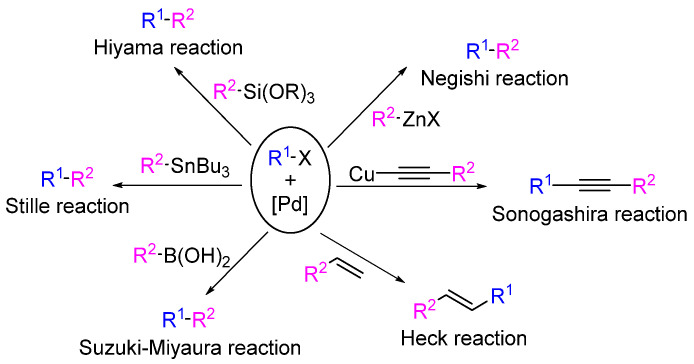
Pd-catalyzed cross-coupling reactions. The fragments that are coupled are indicated by the colors blue and pink.

Among Pd-catalyzed cross-coupling reactions, we find the Suzuki–Miyaura, Negishi and Heck reactions, winners of the Nobel Prize in Chemistry in 2010. In the Suzuki–Miyaura reaction [26,27], arylboronic acids and esters are coupled with aryl halides in the presence of a palladium catalyst to give biaryls. On the other hand, the Negishi reaction [28] consists of a Pd-catalyzed coupling of aryl halides with arylzinc reagents. Meanwhile, the Heck reaction [29] is slightly different with respect to the previous two reactions and other cross-coupling reactions as it does not use any organometallic reagent. This transformation involves aryl, alkenyl and benzyl halides that are directly coupled with an alkene in the presence of a palladium catalyst. The reaction runs in the presence of a base, and the product obtained is the corresponding substituted alkene. Other examples of Pd-catalyzed cross-coupling reactions are the Stille [30], Hiyama [31] and Sonogashira [32] reactions (Scheme 2).

It should be noted that the reactions described above are not exclusively catalyzed by palladium, as other metals can also be used, but palladium, for the aforementioned reasons, is the most common. Therefore, in this section, we will focus on the application of Pd-catalyzed cross-coupling reactions in the synthesis of different marine drugs.

The presence of activating groups that are finally cleaved in the substrates that are susceptible to cross-coupling transformations is against the atom economy and sustainability of the process. Organic halides are commonly used as substrates, which donate the alkyl, aryl or alkenyl substituent. This leads to the formation of HX as waste materials [24], which has a negative impact from a green chemistry point of view. Therefore, in the last years, the interest in palladium-mediated C-H functionalization/activation has highly increased, as it allows the functionalization of C-H bonds even in the presence of more reactive functional groups while decreasing the amount of waste material [33]. 

**Scheme 3:**
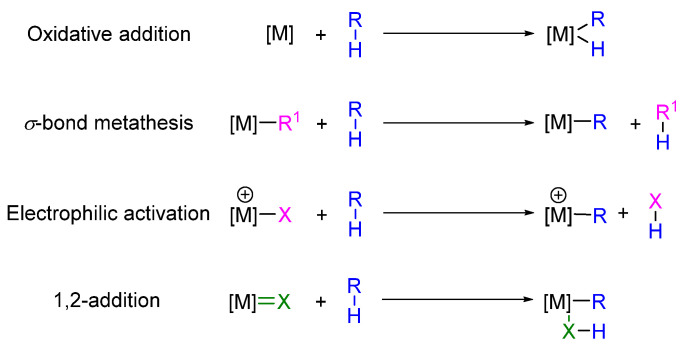
C-H activations’ mechanisms. The R-H bond that is activated is indicated by blue; pink indicates the substituent (R^1^ or X) of the metal that replaces the R substituent of the R-H bond; green indicates the unsaturated M-X bond, in which the R-H bond is added.

In contrast to cross-coupling reactions, for which there was a general mechanism, we can find different mechanisms for C-H activation, dictated by the oxidation state and identity of the transition metal catalyst (Scheme 3). Between them, the most common are oxidative addition, in which an R-H bond is broken to form M-H and M-C bonds; σ-bond metathesis, which involves a hydride or an alkyl complex and involves the breaking of M-R and R^1^-H bonds in favor of the formation of M-R^1^ and R-H bonds; electrophilic addition, in which the metal replaces the hydrogen atom in the substrate, acting as a Lewis acid; and 1,2 addition, in which the C-H bond is added to an unsaturated M-X bond. For palladium, among these mechanisms, the most common are oxidative addition and σ-bond metathesis [33].

In this section, some examples of cross-coupling reactions catalyzed by palladium complexes applied in the synthesis of marine drugs are exposed.

#### 2.1.1. Suzuki–Miyaura Cross-Coupling Reaction

**Scheme 4:**
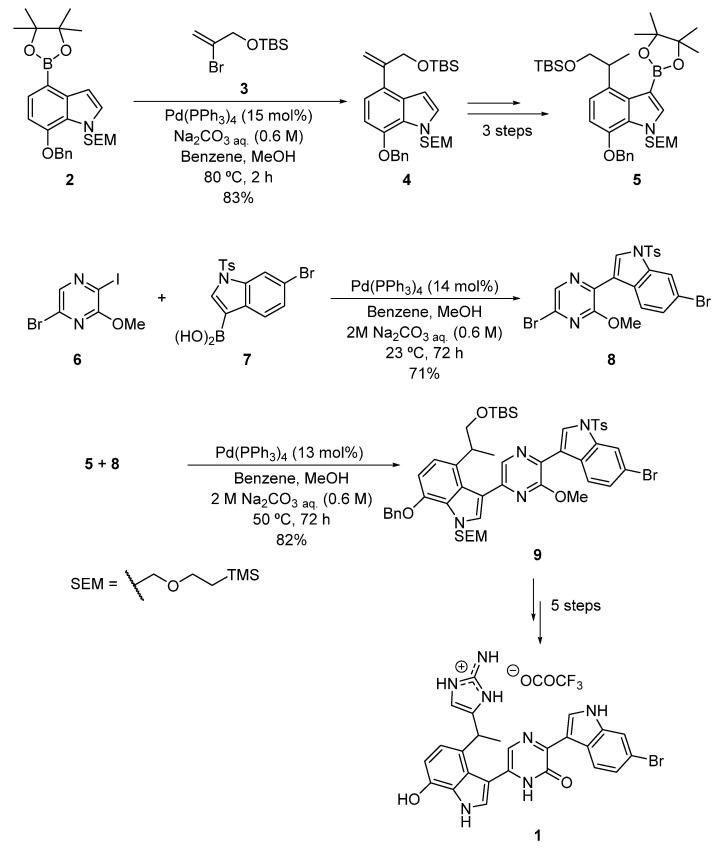
Suzuki–Miyaura reactions in the total synthesis of Dragmacidin D **1**.

Dragmacidins are bisindole alkaloids isolated for the first time in 1988 from the deep-water marine sponge *Dragmacidon* sp. [34]. These marine alkaloids are characterized by a bisindole structure, in which a 6-bromoindole is bonded to a piperazine, pyrazinone or pyrazine ring. Currently, 11 derivatives of dragmacidins are known, and many of them have demonstrated good antitumor, antiviral, cytotoxic, antifungal and antimicrobial activities [35]. Among these natural products, Dragmacidin D **1** is a powerful inhibitor of serine–threonine protein phosphatases. It also induces cytotoxicity in the murine leukemia cell line and in the human lung adenocarcinoma cell line [36]. The biological properties of this compound have prompted the development of synthetic routes to Dragmacidin D and precursors. In 2002, Stoltz et al. [37] reported a total synthesis for this alkaloid in which the key reactions were sequential Pd-catalyzed Suzuki–Miyaura cross-coupling reactions (Scheme 4). In the first of them, the indole derivative **2** reacted in the presence of Pd(PPh_3_)_4_ with vinyl bromide **3** to give indolylether **4** with a yield of 83%. This indolylether **4** was then transformed into fragment **5** after three synthetic steps. In a parallel way, a Suzuki–Miyaura reaction between dihalopyrazine **6** and indole derivative **7** catalyzed by Pd(PPh_3_)_4_ afforded compound **8** in 71% yield. Afterward, a Suzuki–Miyaura cross-coupling reaction using the previously formed fragments **5** and **8**, mediated by the palladium catalyst employed before, yielded the bis-indole alkoxypyrazine **9**. It should be noted that the desired product **9** was obtained with good yields and selectivity under very controlled conditions and the lowest possible temperature (50 °C), as the reaction required good temperature control. If the temperature reached 80 °C, the coupling reaction of the bromoindole unit would become competitive with the targeted one. Finally, five synthetic additional steps were required to access the target molecule Dragmacidin D **1**.

**Scheme 5:**
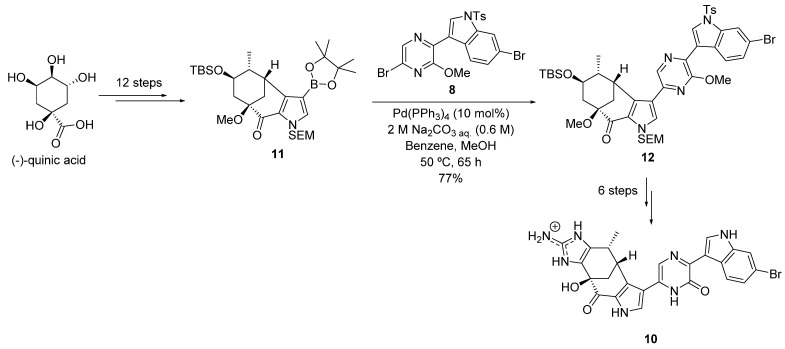
Cross-coupling reactions in the total synthesis of Dragmacidin F **10**.

Another dragmacidin that has attracted attention because of its biological and pharmacological activities is Dragmacidin F **10**, which has shown antiviral activity against the herpes virus and human immunodeficiency virus [38]. Stoltz et al. [39] developed a total synthesis for **10** starting from a single enantiomer of quinic acid (Scheme 5). In this total synthesis, a series of halogen-selective Suzuki cross-coupling reactions were used to synthesize the carbon skeleton of this marine alkaloid. First, the pyrroloboronic ester **11** underwent a halogen-selective Suzuki cross-coupling reaction with the previously shown fragment **8**. In this step, authors took similar conditions used in the analog step on the synthesis of Dragmacidin D **1** (Scheme 5) [37] to obtain the desired product **12** with a 77% yield. Finally, compound **12** was transformed into Dragmacidin F **10** after six more steps.

Dictyodendrins are marine alkaloids isolated from the marine sponge *Dictyodendrilla verongiformis* for the first time in 2003. They are pyrrolo(2,3-*c*)-carbazole-derived compounds that have shown anti-telomerase activity, which is found in 90% of human tumors [40]. In other words, these marine alkaloids are potential antitumor agents; thus, their synthesis has been a topic of interest among chemists.

**Scheme 6:**
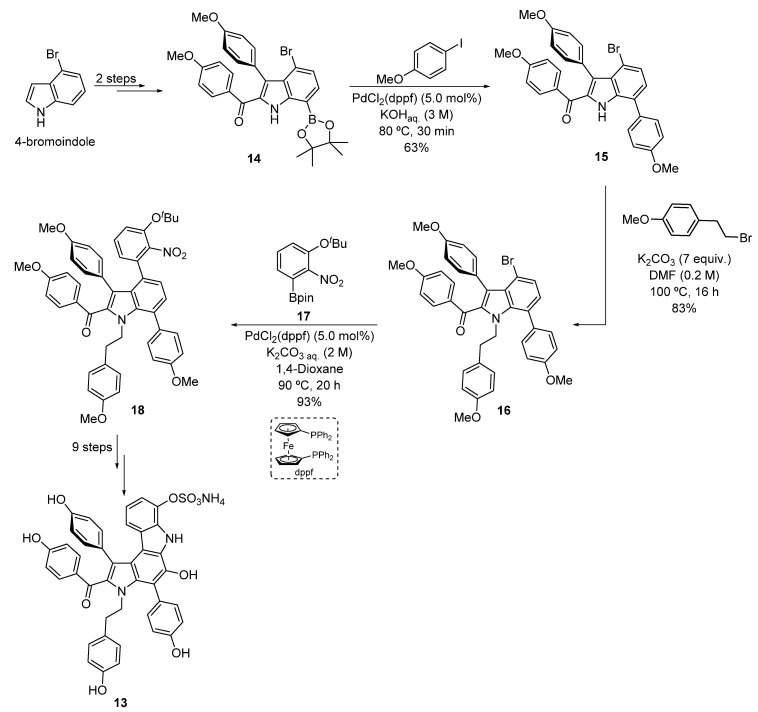
Suzuki–Miyaura reactions in the total synthesis of Dictyodendrin B **13**.

Gaunt et al. [41] proposed a total synthesis of Dictyodendrin B **13** starting from the commercially available 4-bromoindole (Scheme 6). They followed a divergent strategy, which involved the sequential direct functionalization of each position of this central heteroatomic scaffold. In this synthetic pathway, the functionalization of the C-7 position on compound **14**, derived from the 4-bromoindole, was achieved by a Suzuki–Miyaura coupling. Thereby, the cross-coupling reaction between this compound **14** and 4-iodoanisole was performed in the presence of the catalyst PdCl_2_(dppf) and an aqueous solution of KOH, yielding compound **15** with a 63% yield. Similarly, the functionalization of the C-4 bromide was achieved via a Suzuki–Miyaura cross-coupling reaction between compound **16**, obtained from **15** after an N-H alkylation, and the nitrophenol-derived boronic ester **17.** The reaction was performed in the presence of PdCl_2_(dppf) in a mixture of an aqueous solution of K_2_CO_3_ and dioxane and 90 °C. The targeted product **18** was therefore obtained with a yield of 93%. It must be noted that the aryl boronic ester must be added dropwise to avoid the deleterious protodeboronation process that was observed when the ester was present in the reaction mixture from the beginning. Finally, nine additional synthetic steps allowed the authors to obtain the natural marine drug Dictyodendrin B **13**.

Discodermolide **19** is a polyketide isolated for the first time in 1990 from the marine sponge *Discodermia dissolute* [42]. Its structure consists of a 24-carbon backbone with 13 stereocenters, a carbamate moiety, a tetrasubstituted *γ*-lactone and a high degree of unsaturation. This marine compound has shown potent cell growth inhibitory and immunosuppressive activity, and it acts as a microtubule-stabilizing agent and mitotic spindle poison. It has also exhibited cytotoxicity against some cancer cell lines [43].

**Scheme 7:**
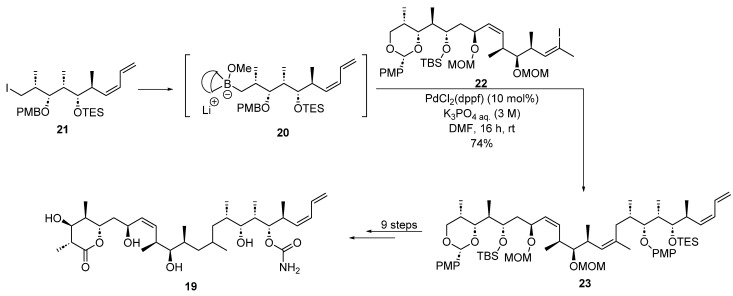
Suzuki reaction in the total synthesis of (+)-Discodermolide **19**.

There are many total syntheses reported for Discodermolide **19**, in which a Pd-catalyzed cross-coupling reaction is employed. Marshall et al. [44] proposed a total synthesis for Discodermolide, in which the last C-C bond-forming reaction required to synthesize the Discodermolide skeleton was a Suzuki coupling reaction between the trialkyl boronate **20**, prepared in situ from iodide **21**, and vinyl iodide **22** in the presence of PdCl_2_(dppf) (Scheme 7). Product **23** was therefore obtained with a yield of 74%. Nine additional steps afforded the desired natural product Discodermolide **19**.

**Scheme 8:**
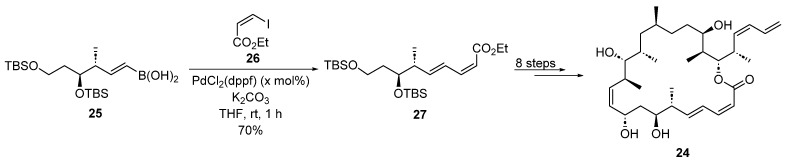
Suzuki reaction in the total synthesis of (−)-Dictyostatin **24**.

Dictyostatin **24** is a polyketide isolated in 1994 from a dark sponge of the genus *Spongia* [45]. This compound can be described as a substituted 22-membered macrolide with 11 stereocenters and a backbone of 26 carbon atoms. Its activity is similar to Discodermolide **19**, and it has shown a low nanomolar cytotoxicity towards different human cancer cell lines [43]. Ramachandran et al. [46] reported in 2007 a total synthesis for this marine polyketide, in which the synthesis of one of its structural subunits included a Suzuki coupling reaction (Scheme 8). In this synthesis, the cross-coupling reaction catalyzed by PdCl_2_(dppf) between boronic acid **25** and (*Z*)-vinyl iodide **26** in THF produced the desired diene ester **27** in 70% yield. The natural product Dictyostatin **24** was finally obtained after eight extra steps.

**Scheme 9:**
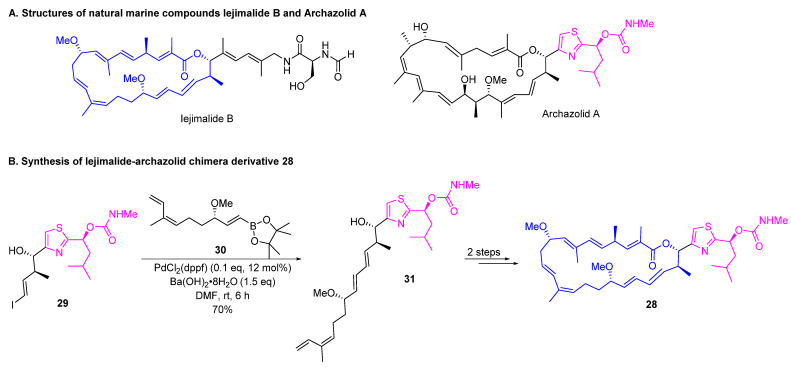
(**A**) Molecular structures of Iejimalide B and Archazolid. The macrolactone ring of Iejimalide B and the side chain of archazolid A are indicated by the colors blue and pink, respectively. (**B**) Suzuki reaction in the synthesis of Iejimalide–archazolid chimera **28**.

Iejimalide B (Scheme 9A) is a marine compound first isolated from the tunicate *Eudistoma* cf. *rigida* [47] that can be described as a substituted and polyunsaturated 24-membered macrolide with anticancer activity and selectivity to NCI 60 cell lines. This marine compound is also a specific inhibitor of v-ATPases [48,49]. On the other hand, Archazolid A (Scheme 9A) is a secondary metabolite derived from a terrestrial myxobacteria of the strains *Archangium gephyra* and *Cystobacter* sp. [50]. As Iejimalide B, this compound is a substituted and polyunsaturated 24-membered macrolide and functions as a potent inhibitor of v-ATPases, which makes it a potential anti-metastatic drug [49,51]. 

The similarities in structure and activity of these compounds motivated Fürstner et al. [49] to design and synthesize an Iejimalide–archazolid chimera **28** containing the macrolactone ring of Iejimalide B and the side chain of Archazolid A. In their approach, the side chain building block **29** reacted with boronate **30** under PdCl_2_(dppf) catalysis, via a Suzuki cross-coupling reaction. Product **31** was obtained with a yield of 70%, and two more steps provided the Iejimalide–archazolid chimera **28** (Scheme 9B).

#### 2.1.2. Heck Cross-Coupling Reaction

Trabectedin **32** is a tetrahydroisoquinoline alkaloid isolated in 1990 from the Caribbean tunicate *Ecteinascidia turbinate* [52]. Its structure consists of three rings and contains a methanolamine center [53]. This marine compound is also known as Ecteinascidin 743 or Yondelis^®^, and it has shown potent cytotoxicity against different tumor cell lines. This anticancer drug was approved by the European Union for the treatment of soft tissue sarcoma in 2007, for the treatment of relapsed platinum-sensitive ovarian cancer in 2007, and it has been prescribed for the treatment of metastatic liposarcoma or leimyosarcoma in the United States since 2015 [53,54].

**Scheme 10:**
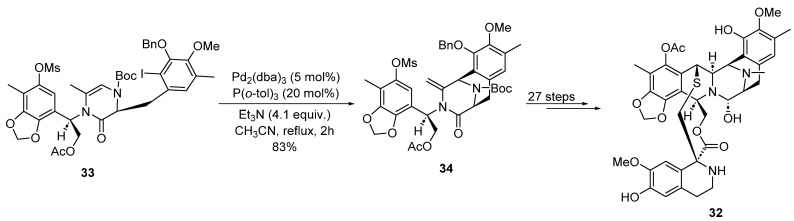
Intramolecular Heck reaction in the total synthesis of Trabectedin **32**.

In 2002, Fukuyama et al. [55] reported a total synthesis of trabectedin, in which an intramolecular Heck reaction catalyzed by palladium was crucial for the synthesis of the bicyclo(3.3.1) system (Scheme 10). Thus, the cyclic enamide **33** was reacted under mild conditions in the presence of Pd_2_(dba)_3_ and P(*o*-tol)_3_ to give the tricycle **34** an 83% yield. The target marine drug could be synthesized by employing 27 additional steps.

Pseudopsterosins are a family of diterpene glycosides isolated in 1985 from *Pseudopterogorgia elisabethae*, a Caribbean Seawhip [56]. Thirty-one different pseupterosins have been identified, and they differ in the grade of acetylation at the sugar ring. These structural differences determine their diverse biological and cytotoxic activities. Nevertheless, all of them have been demonstrated to have potent analgesic, anti-inflammatory, wound-healing and anti-cancer activities, among others, and they can be found in different skin creams and cosmetic products [57,58].

**Scheme 11:**
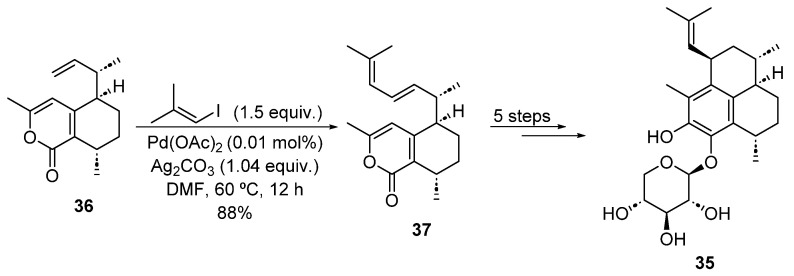
Heck reaction in the total synthesis of Pseudopterosin A **35**.

Among these pseudopterosins, the Pseudopterosin A **35** is one of the most studied. It inhibits phagocytosis and activates calcium release, as well as reduces cellular bursts during cellular stress in unicellular protists [57]. Yu et al. [59] reported a synthetic route to this glycoside, in which one of the key steps consists of a regioselective Heck reaction (Scheme 11). Herein, the α-pyrone **36** was coupled with 1-iodo-2-methylprop-1-ene in the presence of the catalyst Pd(OAc)_2_ and the base Ag_2_CO_3_. The diene **37**, obtained with an 88% yield and an *E*/*Z* ratio over 19:1, could be converted in the final Pseudopterosin A **35** after five additional steps.

#### 2.1.3. Negishi Cross-Coupling Reaction

**Scheme 12:**
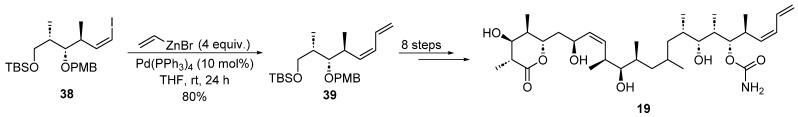
Negishi reaction in the total synthesis of (+)-Discodermolide **19**.

As previously reported, there are many total syntheses for Discodermolide **19**, in which a Pd-catalyzed cross-coupling reaction is employed. In the synthesis reported by Schreiber et al. [60] in 1996 (Scheme 12), a Negishi reaction was used for coupling vinyl iodide **38** with in situ-generated vinyl zinc bromide. The reaction was carried out in the presence of 10 mol% of Pd(PPh_3_)_4_ and using THF as a solvent, and the final product **39** was obtained with an 80% yield. Eight extra steps afforded the product (+)-Discodermolide **19**.

**Scheme 13:**
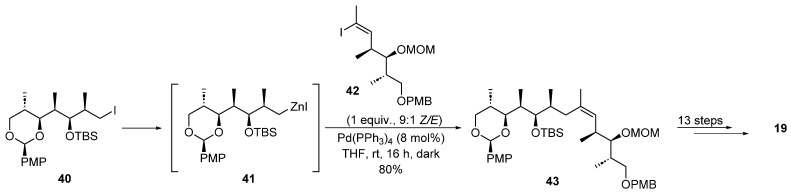
Negishi reaction in the total synthesis of (+)-Discodermolide **19**.

Similarly, Smith et al. [61] developed a synthetic route towards (+)-Discodermolide **19**, in which one of the key fragments’ couplings was performed through a Pd-catalyzed Negishi reaction (Scheme 13). In this reaction, the organozinc species **41** was generated in situ from compound **40**. Then, it reacted with vinyl iodide **42**, using Et_2_O as a solvent and in the presence of Pd(PPh_3_)_4_ to produce the compound **43** with an 80% yield. Target compound (+)-Discodermolide **19** was isolated after 13 additional steps.

**Scheme 14:**
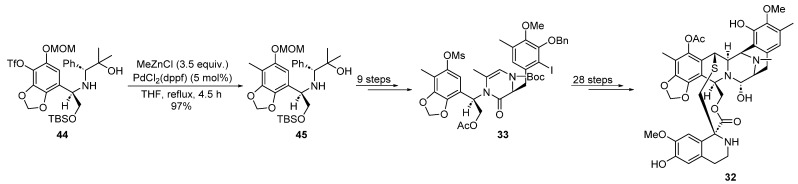
Negishi coupling to access fragment **33** in the total synthesis of Trabectedin **32**.

In the Heck cross-coupling reaction section, the total synthesis of Trabectedin **32** by Fukuyama [55] was mentioned, in which one of the key steps was an intramolecular Heck reaction of **33** (Scheme 10). A Negishi reaction was also a key transformation used previously for the synthesis of one of the constituent fragments (Scheme 14). Thus, the methyl group present in the aromatic ring of **32** was introduced by the Pd-catalyzed cross-coupling reaction of **44** with methylzinc chloride, giving the product **45** with a 97% yield. Nine additional steps were required to obtain intermediate **33**, which was then converted to the final product as explained before.

#### 2.1.4. C-H Activation

**Scheme 15:**
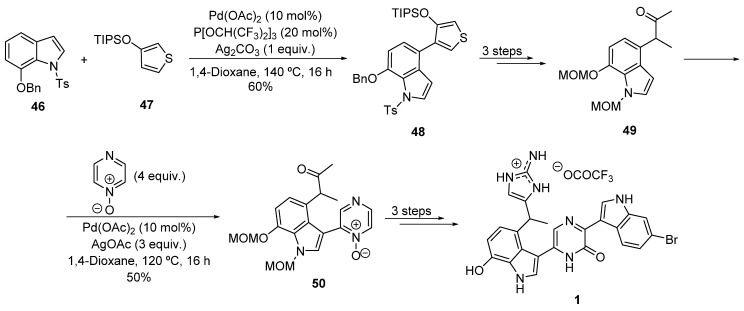
C-H activation reactions in the total synthesis of Dragmacidin D **1** [62].

In previous sections, it has been exposed to Stoltz’s synthesis of Dragmacidin D **1** [37], where the key reactions were sequential Suzuki–Miyaura cross-coupling reactions (Scheme 4). This synthesis, while being effective, required a lot of steps, as both organometallic and organic halides coupled in the cross-coupling reactions required to be prior functionalized. When the C-H activation is considered, these prior steps are not needed, and in this line, Itami et al. [62] developed a total synthesis of Dragmacidin D **1**, in which two of the key steps were metal-catalyzed C-H couplings (Scheme 15). First, the coupling of the indole **46** with 3-triisopropylsilyloxy-subtituted thiophene **47** was conducted using the catalytic system Pd(AcO)_2_/Ag_2_CO_3_/P(OCH(CF_3_)_2_)_3_ and 1,4-dioxane as a solvent, achieving the product **48** with yields of 60% and perfect regioselectivity. Then, the bis-MOM-protected indole **49** obtained from **48** after a few steps underwent a second C-H/C-H coupling reaction with pyrazine N-oxide, using in this case the catalytic system Pd(AcO)_2_/AgOAc. Product **50** was obtained with moderate yields but with perfect chemoselectivity and could be converted into the desired product after three steps.

**Scheme 16:**
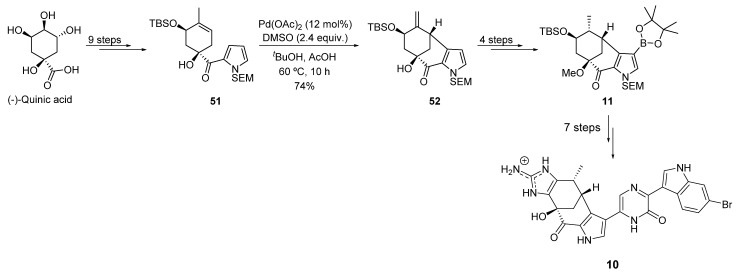
C-H functionalization to access fragment **11** in the total synthesis of Dragmacidin F **10**.

As previously exposed, Stoltz et al. [39] developed a total synthesis for Dragmacidin F **10**, in which its carbon skeleton was synthesized via several Suzuki reactions. In this synthesis, the authors also employed a C-H activation for the synthesis of one of the previous intermediates of **11** (Scheme 16). Thus, a palladium-mediated C-C bond-forming reaction (C-H activation) was employed to obtain compound **52**, starting from pyrrole **51** and using DMSO as a ligand. The desired bicycle was obtained with a 74% yield and as the only stereo- and regioisomer present. After several steps, it was transformed into the pyrroloboronic ester **11**, which was further transformed into product **10** as mentioned before.

#### 2.1.5. Tsuji–Trost Allylation

Tetrodotoxin **53** is a highly functionalized zwitterion isolated in 1990 from the puffer fish *Spheroides rubripes* [63]. It contains an *ortho* acid, a cyclic guanidine and an aminal in its structure, and it is responsible for puffer fish toxicity. This marine compound is known for its ability to inhibit voltage-dependent Na^+^ channels, and this has made tetrodotoxin widely used in neuroscience and neurophysiology [64]. 

**Scheme 17:**
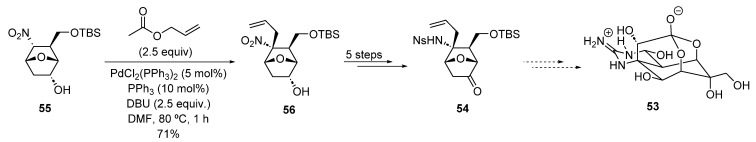
Tsuji–Trost allylation in the total synthesis of tetrodotoxin [64].

Shinada et al. [64] reported a stereoselective synthesis of the key intermediate **54** in the synthesis of Tetrodotoxin **53** (Scheme 17). The Pd-catalyzed Tsuji–Trost allylation [65] of compound **55** was key in the construction of the quaternary amino carbon center on the bicyclic derivative **55**. This transformation employed PdCl_2_(PPh_3_)_2_ as catalysts and allyl acetate as an allyl source, rendering intermediate **56** in a 71% yield. The compound **56** was then transformed into oxanorbornene **54**, which could be transformed into Tetrodotoxin **53** after several synthetic procedures.

### 2.2. Olefin Metathesis

The metathesis reaction is a useful method for creating new C-C bonds, along with the Wittig, Diels–Alder and Grignard reactions and palladium-catalyzed cross-coupling reactions, which are four of the most important discoveries that have made significant progress in organic synthesis [66].

Derived from the work of Grubbs [67], the ruthenium-derived catalysts have opened up new avenues for the efficient construction of complex molecular architectures. Specifically, the olefin metathesis reaction has led to breakthroughs in the development of biologically active compounds and new drugs, as well as industrial products and polymers, resulting in Y. Chauvin, R. H. Grubbs and R. R. Schrock winning the Nobel Prize in 2005 [68].

**Scheme 18:**
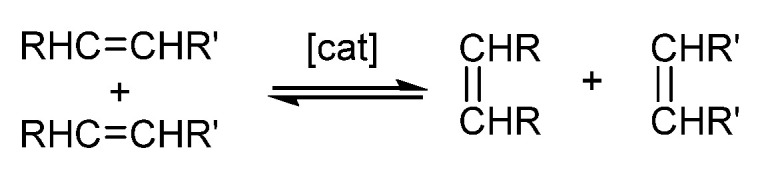
General catalyzed olefin metathesis reaction.

This reversible reaction is based on a transition-metal-catalyzed process, where olefins rearrange their bonds for a redistribution of their carbon skeleton as shown in Scheme 18 [69].

**Figure 1:**
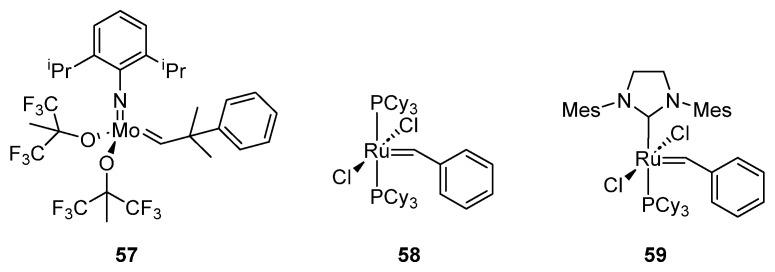
Schrock’s catalyst **57**, first-generation Grubbs’ catalyst **58** and second-generation Grubbs’ catalyst **59**.

This methodology is usually catalyzed by metal carbene complexes. The Schrock catalyst and the first- and second-generation Grubbs catalysts are commercially available, and they are commonly used (Figure 1). Ruthenium catalysts are less active than Molybdenum catalysts, although they have shown excellent functional group tolerance [66].

Olefin metathesis reactions offer numerous advantages in organic synthesis, including high atom economy, mild reaction conditions and huge compatibility with a large range of functional groups and enable access to biologically relevant compounds [70]. Thus, the versatility of metathesis reactions transcends some synthetic limitations, by offering many opportunities in organic synthesis [67]. The main transformations based on the metathesis reaction are presented in Table 1.

These methodologies have facilitated numerous synthetic routes for the preparation of marine drugs with anticancer activity. The selected examples shown afterward are mostly based on type 1, 2 and 3 metathesis reactions.

#### 2.2.1. Cross-Metathesis (CM): Type 1 Reaction

**Figure 2:**
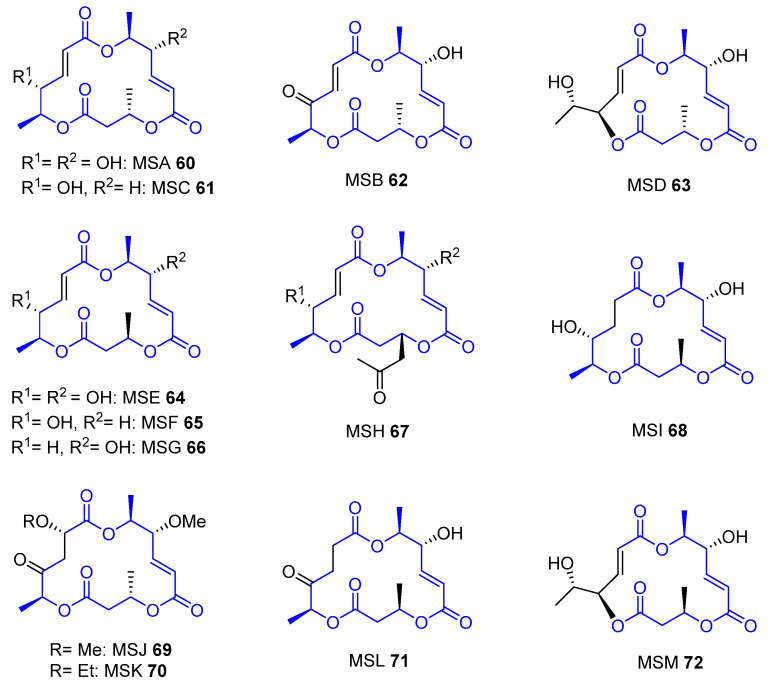
Molecular structures of compounds from the Macrosphelide (MS) family, where the blue color represents the core of these macrolides [72].

A promising strategy to design new anticancer agents consists of the conjugation of natural marine products with other biologically active agents with a linker. The addition of linkers should not influence the biological performance of the chemotherapeutic payload, should not induce aggregation and limit the premature release of payloads in plasma and effectively release active molecules at targeted action sites. A good example of such is the macrosphelide A-biotin and avrainvillamide–biotin chimeras’ case [71]. It has been designed to identify both the target protein and the mechanism of action used by the macrosphelide family (Figure 2). Macrosphelide A is a natural polyketide with multiple biological properties, such as its anticancer activity against lung metastasis of B16/BL6 melanoma in mice or the inhibition of human leukemia HL-60 cells adhesion to the monolayer of lipopolysaccharide-activated human-umbilical-vein endothelial cells (HUVECs) [72]. It was isolated for the first time from the fermentation broth of *Micreosphaeropsis* sp. FO-5050 and later from a mycoparasite (*Coniothyrium minitans*) and a fungus (*Tritirachium* sp. HKI 0317) [73].

**Scheme 19:**
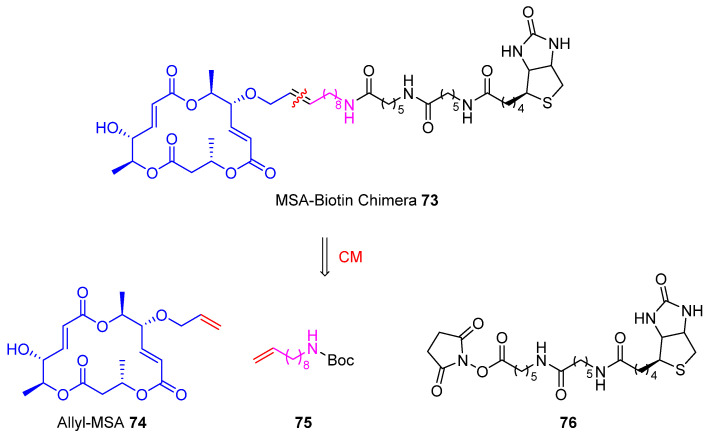
MSA–biotin chimera retrosynthesis, where blue and pink colors represent the different fragments, and the red color shows the double bond participating in the metathesis.

Yun et al. synthesized the MSA–biotin chimera **73** with different fragments as shown in Scheme 19. Although some steps were required to obtain these molecules, the ruthenium-catalyzed step took place between the allyl **74** and the Boc-protected alkene linker **75**, in which the amino group was protected by Boc [69].

**Scheme 20:**
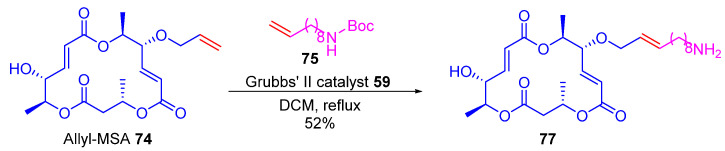
CM reaction for the synthesis of MSA–biotin chimera precursor **74**, where blue and pink colors represent the different fragments, and the red color shows the double bond participating in the metathesis.

As shown in Scheme 20, the reaction between fragments **74** and **75** is a type 1 metathesis produced by the second-generation Grubbs’ catalyst **59**, in dichloromethane as a solvent and under reflux to generate **77** in 52% yield. Subsequent deprotection of the amine and biotinylation with **76** produced the final MSA–biotin chimera **73** [73]. Ongoing research is focused on targeting fishing studies, using this chimera, to investigate a potent inhibitor of cell–cell adhesion [71].

Avrainvillamide was used to synthesize another example of biotin chimeras. It is a natural product isolated from *Aspergillus* sp. CNC358 and with antiproliferative properties in numerous human cancer cell lines. The biotin conjugate is applied within an efficient method to identify and isolate potential protein-binding partners from cancer-cell lysates [74]. In this case, the nucleophosmin (a multifunctional protein in numerous tumors) was recognized as the target protein of avrainvillamide, after Western blotting and MS/MS sequencing of this chimera-treated T-47 D cells [71].

**Scheme 21:**
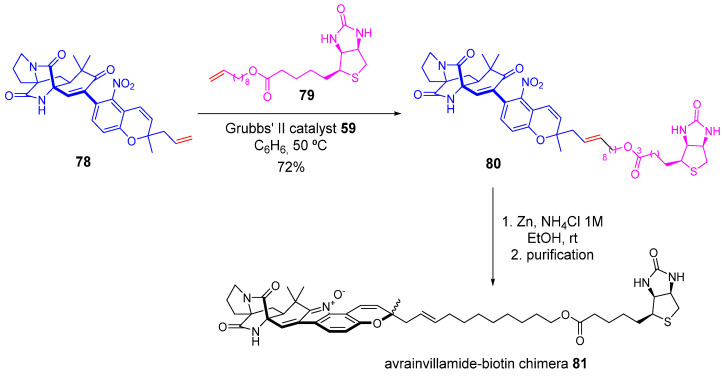
CM reaction on the synthesis of avrainvillamide–biotin chimera **81**, where blue and pink colors represent the different fragments, and the red color shows the double bond participating in the metathesis.

The type 1 olefin metathesis reaction reported in the synthesis of the avainvillamide–biotin chimera 81 is depicted in Scheme 21. A reaction between intermediate 78 and the terminal alkene of 79 catalyzed by 59 occurred in benzene at 50 °C giving rise to intermediate 80 in a 72% yield. A subsequent step was a reductive cyclization of 80, followed by HPLC purification, leading to the target molecule 81 [71].

#### 2.2.2. Ring-Closing Metathesis (RCM): Type 2 Reaction

**Figure 3:**
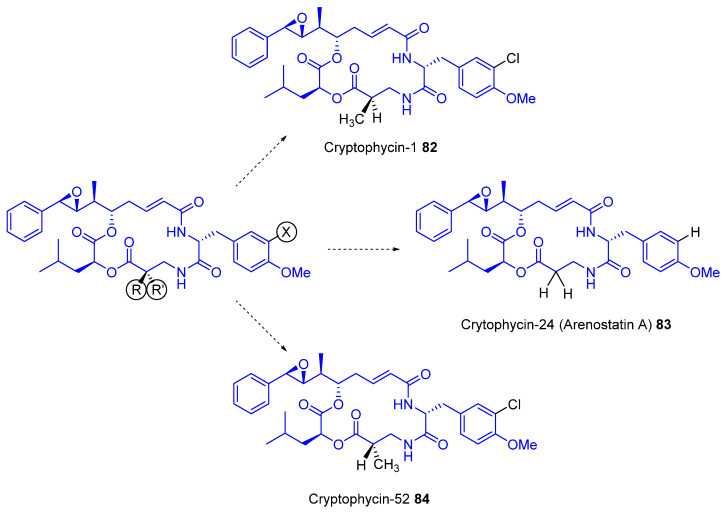
Cryptophycin structures, where the blue color shows the part of the compound that remains unchanged [75].

Arenostatin A **83** is a marine drug that belongs to the cryptophycin family, which consists of a group of 16-membered macrolides (Figure 3), with great importance in the oncology field as a new promise of anticancer agents [53]. 

The depsipeptide **83** was elucidated by Kobayashi et al., and it is derived from the Okinawan Marine Sponge *Dysidea arenaria*. This compound displays extremely potent cytotoxicity against KB cells, with an IC_50_ of 5 pg/mL [76]. Additionally, the same molecule was subsequently found in a blue-green alga, *Nostoc* sp. GSV 224, and was named Cryptophycin 24, with significant inhibition in vitro of tubulin assembly [77].

**Scheme 22:**
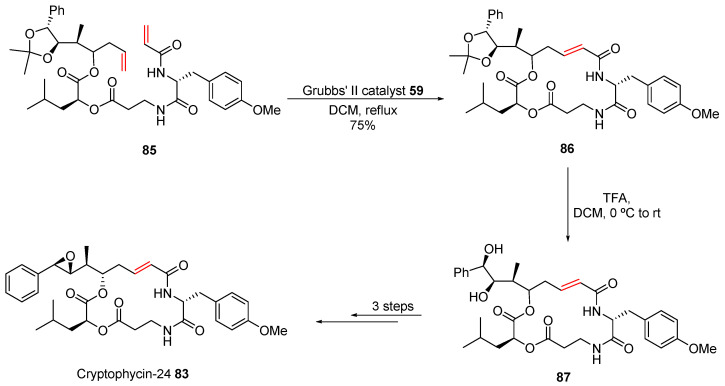
Ruthenium-catalyzed step from cryptophycin-24 synthesis [78], where red color shows the double bond participating in the metathesis.

Yadav et al. developed a complete Cryptophycin-24 synthesis via Prins cyclization, in which there is a step where intermediate **85** is transformed into compound **86** through an RCM metathesis catalyzed by the second-generation Grubbs’ catalyst **59** (Scheme 22). The reaction took place under reflux conditions for 2 h using dichloromethane and generating the product in a 75% yield [78].

**Figure 4:**
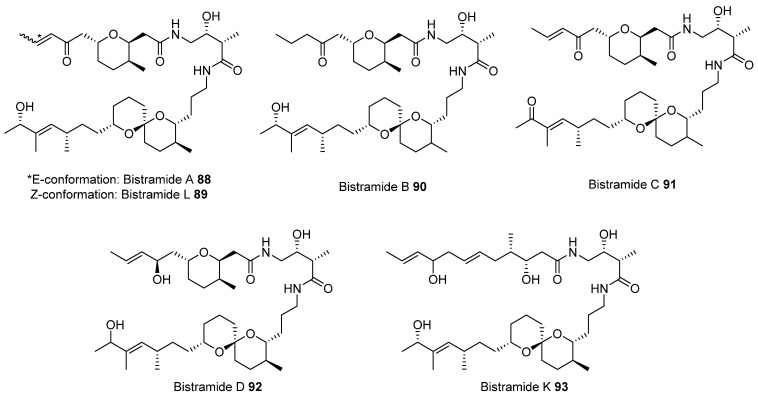
Molecular structure of compounds from the Bistramide family [80].

Another outstanding marine product is Bistramide A **88**. It is a macrocyclic peptide that belongs to the Bistramide family (Figure 4), a class of natural products characterized by their complex macrocyclic structure and potent bioactivity [79].

Bistramides A, B, C, D, K and L are part of a group of natural compounds obtained from a marine organism known as an ascidian, specifically *Lissoclinum bistratum* [81]. Biard et al. isolated and elucidated the structure of Bistramides A-D and K (Figure 4). These molecules showed cytotoxic activity in vitro toward six tumor cell lines [79]. Moreover, Bistramides also displayed neurotoxic, antiparasitic, immunomodulatory and antiproliferative effects [80].

**Scheme 23:**
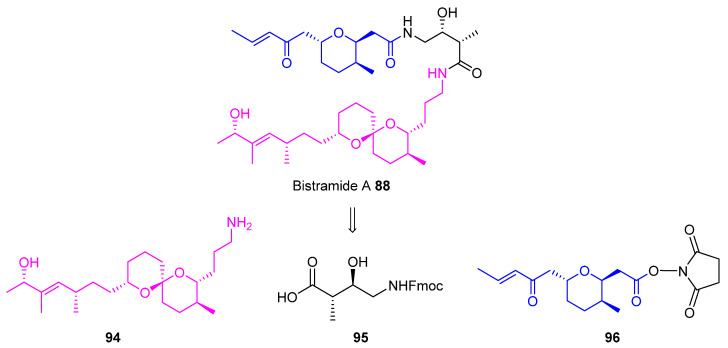
Bistramide A retrosynthesis, where blue and pink colors represent the different fragments.

Because of the aforementioned biological properties, there are compelling reasons to develop synthetic routes for this family of marine drugs. In particular, Bistramide A stands out for its potent antiproliferative effects: Kozmin and coworkers reported how this highly cytotoxic marine metabolite used the actin protein as a receptor by altering its cytoskeleton and inhibiting its polymerization. They also showed that Bistramine A exhibits strong antiproliferative properties in various types of cancer cells, with concentrations leading to 50% growth inhibition (GI_50_) ranging from 20 to 45 nanomolar values [81]. Moreover, the same research group developed successfully Bistramide A synthesis by reaction of fragments **94**–**96** (Scheme 23).

**Scheme 24:**
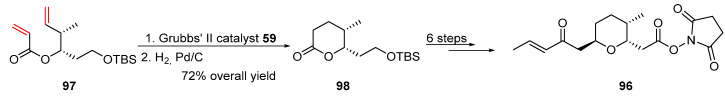
Ruthenium-catalyzed steps in fragment **96** synthesis, where red color shows the double bond participating in the metathesis.

The synthesis of fragment **96** contains a ruthenium-catalyzed step, consisting of an intramolecular type 2 metathesis reaction catalyzed by **59** (13 mol%). A subsequent catalytic hydrogenation gave rise to tetrahydropyran **98** with a yield of 72%. Then, through 6 additional steps, **96** was obtained stereoselectively with a 46% yield (Scheme 24) [82].

#### 2.2.3. Ring-Opening Cross-Metathesis (ROCM): Type 3 Reaction

**Scheme 25:**
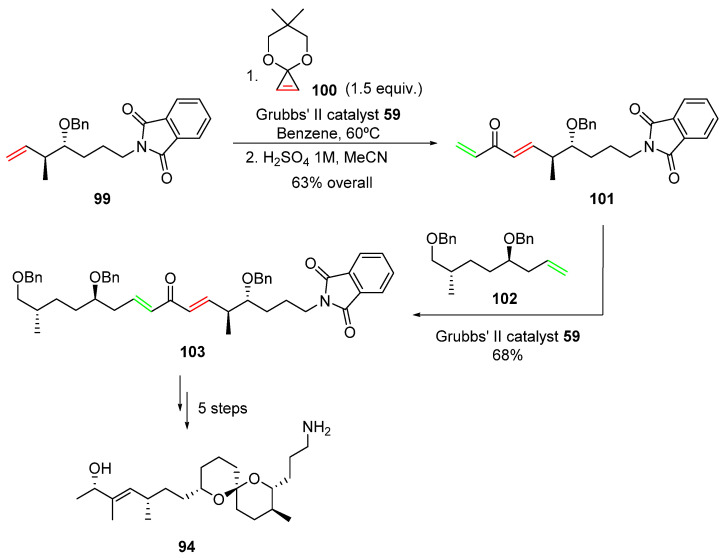
Ruthenium-catalyzed steps in intermediate **94** synthesis, where red color shows the double bond participating in the ROCM metathesis and green color shows the double bond participating in the CM metathesis.

The synthetic route of **94**, a key molecule according to the retrosynthetic planning of Bistramide A (Scheme 23), started with a ruthenium-catalyzed type 3 reaction catalyzed by **59** (10 mol%), between alkene **99** and the cyclopropene acetal **100.** The reaction took place in benzene at 60 °C. After removing the acetal in acidic conditions, dienone **101** was obtained in 63% yield. Subsequently, it was treated with **102** and the previous catalyst **59** leading to a CM metathesis to obtain compound **103** in 68% yield. An ulterior 5-step sequence rendered stereoselectively intermediate **94**, with a 40% yield (Scheme 25), and allowed to prepare Bistramide A in subsequent reactions [82].

### 2.3. Gold Catalysis in the Total Synthesis of Marine Drugs

Once referred to as an inert element, gold has defied expectations in recent decades emerging as an important tool for organic chemists in increasing molecular complexity [83]. Since the pioneering work by Ito and Hayashi [84], gold catalysis has experienced exponential growth in the so-called “gold rush” [85]. Nowadays, this field still represents a burgeoning area of research, in which novel and significant advances are frequently achieved. This development is fueled by several key attributes, including exceptional functional group tolerance, superior biocompatibility, more stability against bleaching and poisoning, high atom economy and frequently distinct reactivity profiles compared to other transition metal catalysts [83]. The employment of gold catalysts in total synthesis has not been left behind, since these coveted features align closely with the needs of this particular area.

Various authors have contributed to compiling the array of gold-catalyzed transformations utilized in synthesizing numerous natural products, shedding light on novel retrosynthetic disconnections and paving the way for innovative approaches [83,86,87]. It is worth noting that the properties of gold catalysts are heavily influenced by their oxidation state. Gold exists in two stable oxidation states, +1 and +3, each exhibiting distinct behavior when interacting with organic molecules. While significant advancements are being made in the field of gold(III) catalysis [87], gold(I) remains predominant, being the most commonly employed species in total synthesis [88,89]. Thus, the evolution of gold catalysis has enabled the development of a broad spectrum of intramolecular and intermolecular transformations, including bis-spiroketalization, hydroalkoxylation, hydrocarboxylation, hydroamination, hydroarylation, oxidation and glycosylation reactions of particularly alkynes, as well as intriguing cycloisomerizations and rearrangements of enynes [83]. 

In the next paragraphs, a collection of gold-catalyzed steps in the total synthesis of biologically active marine natural products has been disclosed, classifying them according to the type of transformation displayed.

#### 2.3.1. Addition of O-/N-Nucleophiles to Alkynes and Allenes

**Scheme 26:**
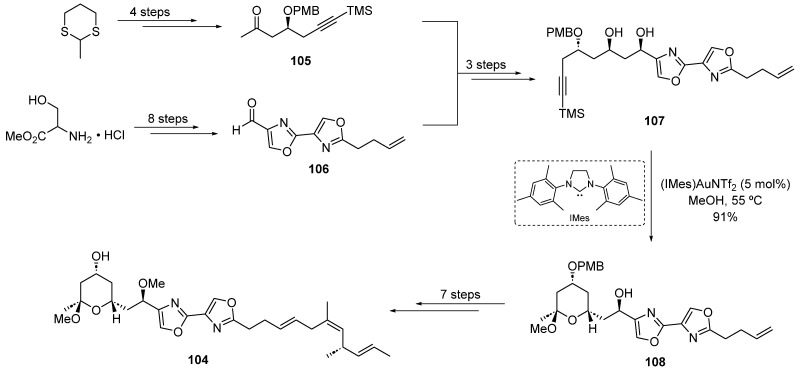
Au(I)-catalyzed ketalization step in the total synthesis of (−)-Hennoxazole A **104**.

Interestingly, the gold(I)-catalyzed hydroalkoxylation of an alkyne can also lead to the formation of ketals in the presence of a second nucleophilic hydroxyl group, both inter- and intramolecularly. This transformation has been exploited in the total synthesis of (−)-Hennoxazole A **104**. Isolated from a species of *Polyfibrospongia* sponge, this marine natural bisoxazole product displayed antiviral activity against herpes simplex 1 and peripheral analgesic activity [90]. The most efficient approach to synthesizing this marine natural product was reported by Ley and coworkers in 2013, employing batch and flow methodologies [91]. The starting material employed in the gold-catalyzed hydroalkoxylation step **107** was crafted through the combination of fragments **105** and **106**, involving a total of 12 synthetic steps (4 for **105** and 8 for **106**). A screening of various gold catalysts and conditions demonstrated that the utilization of 5 mol% of (IMes)AuNTf_2_ in MeOH at 55 °C yielded a remarkable 91% of a single diastereomer **108** (Scheme 26). Remarkably, this intramolecular hydroalkoxylation of a relatively accessible alkynol precursor **107** allowed the construction of the highly functionalized tetrahydropyranyl ring in a stereoselective manner in one step. Subsequently, seven additional steps facilitated the assembly of (−)-Hennoxazole A **104**. 

**Scheme 27:**
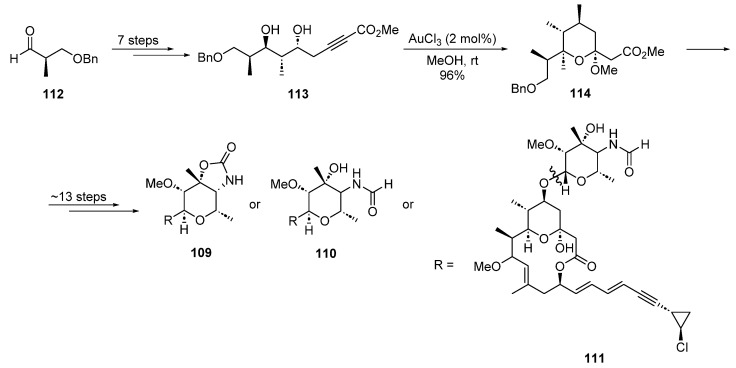
Au(III)-catalyzed ketalization step in the total synthesis of Callipeltosides A–C **109**–**111**.

Another example of a catalytic ketalization of an alkyne precursor can be found in the total synthesis of the Callipeltosides family. These polyketide natural products were isolated as minor metabolites from shallow water lithistida marine sponge *Callipelta* sp. and represent an interesting target due to their cytotoxic activity against human cancer cell lines [92,93]. Ley and coworkers designed a highly convergent approach for efficient preparation of all known members of this class of natural compounds Callipeltoside A-C **109**–**111** [94]. The key step consists of AuCl_3_-catalyzed cycloketalization of intermediate **113**, prepared in seven steps from aldehyde **112**, to form the highly functionalized tetrahydropyran core (Scheme 27). Noteworthy, intermediate **114** was obtained with total selectivity in 96% yield under mild conditions. From this compound **114**, Callipeltosides A-C **109**–**111** can be synthesized in 13 additional reaction steps. This approach represented the first described total synthesis of Callipeltoside B **110**.

**Scheme 28:**
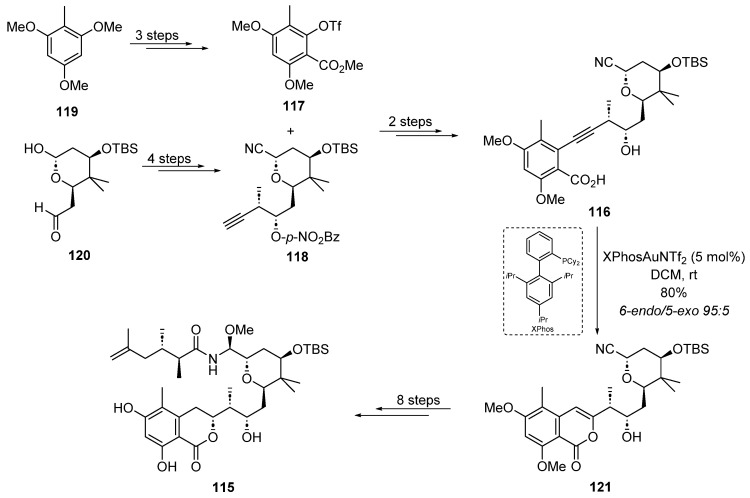
Au-catalyzed hydrocarboxylation step in the total synthesis of Psymberin **115**.

Alkynoic acids have been also exploited under gold catalysis for the construction of (benzo)pyranone rings. An example of this transformation can be found in the total synthesis of Psymberin (or Irciniastatin) **115**. This natural compound **115** was isolated in 2004 from two different marine sponges [95,96], although this complete assignment could not be performed at the time. Due to the potential applications of **115** as a cytotoxic agent against different human cancer cell lines [96], various authors revisited its total synthesis. However, only De Brabander and coworkers were able to selectively obtain this compound in 2012, allowing its full assignment [97]. Their approach contains an intramolecular gold(I)-catalyzed hydrocarboxylation step to form the isocroman-1-one scaffold from an alkyne-containing benzoic acid **116** (Scheme 28). Precursor **116** was convergently synthesized starting from fragments **117** and **118**, obtained from **119** in three steps and **120** in four steps, respectively. Then, the employment of 5 mol% XPhosAuNTf_2_ in CH_2_Cl_2_ at room temperature afforded isocromanone **121** in 80% yield with excellent selectivity (6-endo/5-exo 95:5) (Scheme 28). Remarkably, other Brønsted acid or Lewis acids such as AgSbF_6_ or Zeise’s dimer [Pt(CH_2_CH_2_)Cl_2_]_2_ afforded lower yields and worse regioselectivities, demonstrating the ability of gold catalysis to overcome other transition metal carbophilic catalysis. From intermediate **121**, Psymberin **115** was efficiently synthesized in eight additional steps.

**Scheme 29:**
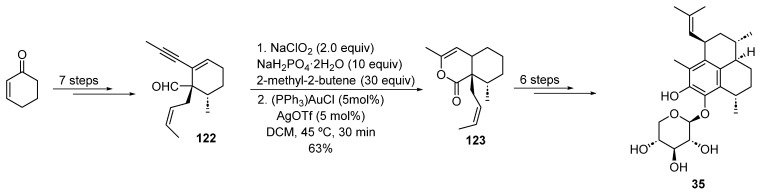
Au-catalyzed hydrocarboxylation steps in the total synthesis of Pseudopterosin A **35**.

The same approach has been employed as a key step in the total synthesis of Pseudopterosin A **35**, previously mentioned in Section 2.1.2. Luo and coworkers reported a synthetic approach employing two different gold-catalyzed steps [59]. The first of them consists of an alkyne hydrocarboxylation reaction after in situ oxidation (Scheme 29). Compound **122** can be obtained in seven linear steps from commercially available cyclohexenone. Then, Pinnick oxidation with NaClO_2_ of aldehyde **122** to its corresponding carboxylic acid followed by treatment with 5 mol% of a 1:1 mixture of PPh_3_AuCl/AgOTf in DCM at 45 °C afforded product **123** in 63% yield in only 30 min. Compound **123** was next employed in a gold-catalyzed step that will be further disclosed in this section, and then six additional steps allowed them to obtain the desired product Pseudopterosin A **35**.

**Scheme 30:**
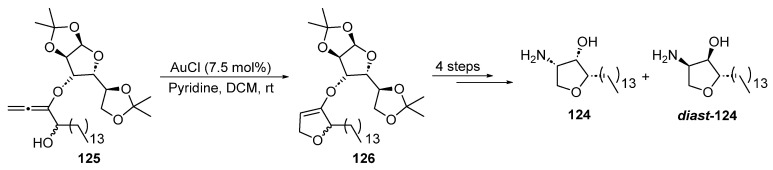
Gold-catalyzed hydroalkoxylation step in the total synthesis of Jaspine B **124**.

Allenes containing strategically placed hydroxyl groups are also intriguing scaffolds for the construction of *O*-heterocycles employing gold catalysis. In this regard, Jaspine B **124**, a natural product bearing isolated in 2002 from the marine sponge *Pachastrissa* sp. with an interesting anticancer activity [98], could be synthesized through a methodology involving the gold(I)-catalyzed hydroalkoxylation of an allene **125** (Scheme 30) [99]. The authors obtained precursor **125** as a diastereoisomeric mixture (*d.r.* 57:43) and then isomerized it in the presence of 7.5 mol% AuCl and pyridine as an additive in CH_2_Cl_2_ to furnish the desired intermediate **126** (31%), along with its diastereoisomer (41%). Other types of base catalysis were tested for this step, proving less efficiency than the gold species. The minor isomer can be separated by HPLC and converted into Jaspine B **124** and its diastereomer ***diast*-124** (40:60 mixture) in four additional steps. 

**Scheme 31:**
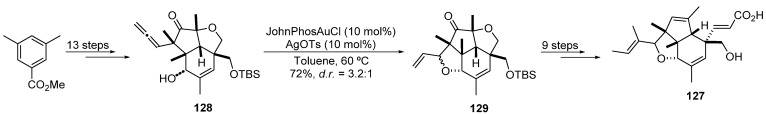
Au-catalyzed hydroalkoxylation step in the total synthesis of Indoxamycin B **127**.

Indoxamycin B **127** a potent antitumor polyketide isolated from saline cultures of marine-derived actinomycetes [100] presents a characteristic, densely functionalized hydroindeno[7,1-*bc*]furan moiety that has been also accessed by hydroalkoxylation of allene intermediate **128** in the presence of gold(I) complexes. Carreira and coworkers [101] reported that precursor **128**, obtained in 13 steps including a gold-catalyzed rearrangement that will be later discussed, was cyclized using 10 mol% JohnPhosAuCl/AgOTs in toluene at 60 °C to afford intermediate **129** as an inseparable 3.2:1 mixture of diastereomers in 72% yield (Scheme 31). After 10 more synthetic steps, the authors were able to achieve compound **127** from species **129** in nine linear steps.

**Scheme 32:**
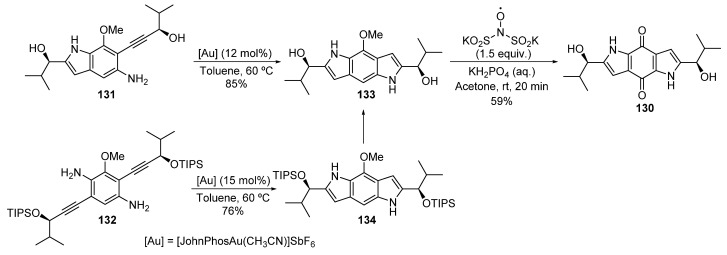
Gold-catalyzed hydroamination step in the total synthesis of (+)-Terreusinone **130**.

The gold-catalyzed addition of *N*-nucleophiles has been also exploited in total synthesis for the construction of *N*-heterocyclic scaffolds. In this context, (+)-Terreusinone **130**, a dipyrrolobenzoquinone analog first isolated in 2003 from the marine fungus Aspergillus terreus [102], can be obtained employing this kind of transformation. The most intriguing part of (+)-Terreusinone **130** is the dipyrrolobenzoquinone core, responsible for its high UV-A protecting ability [102]. There are few methodologies that can afford this motif, all of them suffering from major drawbacks such as the impossibility of working with chiral substrates. However, Sperry and Wang were able to circumvent this limitation by means of gold(I) catalysis [103]. *o*-Alkynylanilines **131** and **132** for the gold-catalyzed step can be easily obtained in seven or five synthetic steps, respectively. Under similar conditions, using 12 or 15 mol% JohnPhosAu(NCMe)SbF_6_ in toluene at 60 °C, dipyrrolo[2,3-*f*]indoles **133** and **134** were obtained in 85 and 76% yield, respectively (Scheme 32). One additional deprotection step transformed compound **134** into compound **133**, which afforded the desired (+)-Terreusinone **130** with an oxidation step. It is worth mentioning that other envisioned Pd-catalyzed reactions such as Larock annulation could not furnish the desired intermediate **133**.

**Scheme 33:**
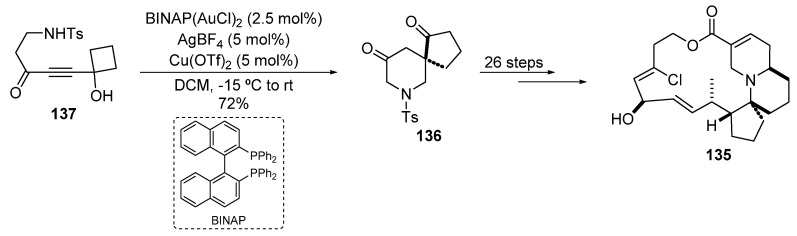
Au(I)/Cu(II)-co-catalyzed hydroalkoxylation-semipinacol step in the total synthesis of (+)-Halichlorine **135**.

Another interesting example was reported for the total synthesis of (−)-Halichlorine **135**. This natural product was isolated from the marine sponge *Halichondria okadai* and is an inhibitor of VCAM-1 expression [104]. It presents a macrolactone with a challenging synthesis of a quinolizine core bearing a spirocyclopentane moiety. Tu, Wan and coworkers devised piperidine derivative **136** as a main intermediate [105]. The key step for the synthesis of piperidine **136** consisted of a gold(I)-copper(II) co-catalyzed tandem hydroamination-semipinacol process, depicted in Scheme 33. Starting from amino alkynone **137**, the use of 2.5 mol% BINAP(AuCl)_2_, 5 mol% AgBF_4_ and 5 mol% Cu(OTf)_2_ as Lewis-acids in CH_2_Cl_2_ at 15 °C afforded the desired product **136** in 72% yield. Noteworthy, the absence of Cu(OTf)_2_ dramatically decreased the yield, while other transition metal catalysts such as PtCl_2_ or Cu(OTf)_2_ alone did not perform this transformation. Finally, (−)-Halichlorine **135** could be obtained after 26 additional steps.

#### 2.3.2. Gold-Catalyzed Cycloisomerization and Cycloadditions

**Scheme 34:**
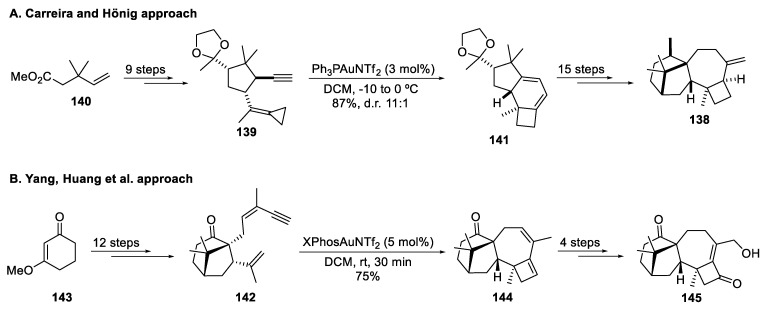
Gold-catalyzed approaches in the total synthesis of Harziane derivatives. (**A**) Carreira and Hönig approach [108]. (**B**) Yang, Huang et al. approach [109].

Gold-catalyzed C-C bond-forming reactions employing *C*-nucleophiles have been also reported in the bibliography, being 1,n-enynes cycloisomerization and alkyne–alkene cycloaddition the most studied one [106]. These particular transformations have been used to access Harzianes through two different pathways. Firstly isolated from *Trichoderma asperellum* fungi collected from the marine red alga *Gracilaria verrucosa*, this family of compounds presents a characteristic [6-5-7-4] fused carbocyclic core with multiple stereocenters. From a therapeutic point of view, they exert interesting anti-HIV, antifungal, anti-inflammatory, antibacterial and cytotoxic activity [107], being therefore interesting scaffolds for drug research. However, their total syntheses are usually quite challenging. On the one hand, Carreira and Hönig reported the total synthesis of a Harziane diterpene **138** by exploiting the gold-catalyzed cycloisomerization of 1,5-enyne **139** with a methylenecyclopropane unit (Scheme 34A) [108]. Starting from ester **140**, the key intermediate **139** was obtained in nine steps. Then, the use of Ph_3_PAuNTf_2_ in DCM afforded the bicyclic compound **141**, with a bicyclo[4.2.0]octane unit, in 87% yield with high diastereoselectivity (dr = 11:1). The final product **138** was obtained after additional 15 reaction steps. On the other hand, Yang, Huang and coworkers employed the gold-catalyzed [2+2] cycloaddition of compound **142** in their synthetic approach (Scheme 34B) [109]. Compound **142** could be obtained in 12 linear steps from **143**. Then, the use of 5 mol% of JohnPhosAuNTf_2_ in DCM at room temperature afforded the key intermediate **144** in 75% yield, with total diastereoselectivity and in only 30 min of reaction. Four additional steps allowed the authors to isolate Harziane diterpenoid derivative **145**.

**Scheme 35:**
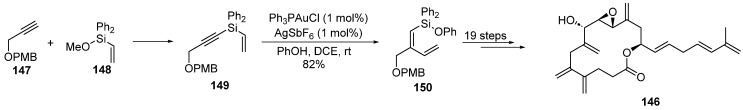
Gold-catalyzed cycloisomerization in the total synthesis of (−)-Amphidinolide V **146**.

The cycloisomerization of 1,n-enynes also offers huge synthetic versatility through the addition of different heteronucleophiles to the intermediate organoaurated species [83]. This reactivity has been employed in the total synthesis of (−)-Amphidinolide V **146**, a macrolactone natural product isolated from the marine plankton *Amphidinium* strain Y-5. Its synthesis is not only methodologically challenging but also interesting from a drug discovery point of view, since **146** and other members of his family exert promising cytotoxicity against L1210 murine lymphoma and human KB carcinoma cells [110]. Lee and coworkers performed the synthesis of (−)-Amphidinolide V **146** by means of a gold-catalyzed cycloisomerization in the early stages of the approach [111]. Starting from readily available **147** and **148**, the starting material **149** for the gold-catalyzed step can be prepared in one step. Treatment of compound **149** in the presence of phenol with a 1 mol% of a 1:1 Ph_3_PAuCl/AgSbF_6_ mixture in 1,2-DCE at room temperature furnished compound **150** in 82% yield (Scheme 35). Additional 19 steps with other fragments synthesized in parallel afforded (−)-Amphidinolide V **146** in 33% overall yield.

#### 2.3.3. Other Gold-Catalyzed Rearrangements

**Scheme 36:**
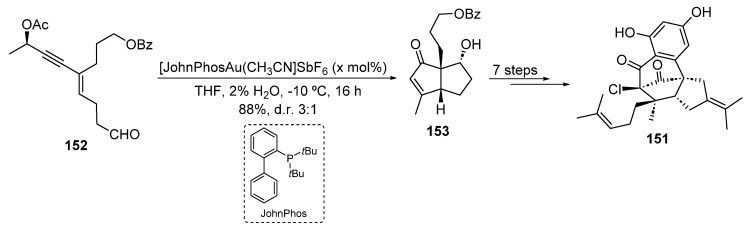
Gold-catalyzed cycloisomerization via propargyl acetate migration in the total synthesis of Merochlorin A **151**.

Gold-catalyzed rearrangements of propargyl acetates through [1,2] or [1,3] acetoxy migrations have been well-documented [83]. An example of this transformation has been reported as a key step in the total synthesis of (−)-Merochlorin A **151**. These natural meroterpenoids were extracted from a marine-based actinomycete strain CNH189, and its main feature consists of an extremely packed tetracyclic bicycle[3.2.1]octadione skeleton. From a therapeutic point of view, (−)-Merchlorin A **151** showed antibacterial activity against methicillin-resistant Staphylococcus aureus (MRSA) and dankastatin C [112]. Given the endemic nature of MRSA in hospitals, a total synthesis that circumvented its tedious and challenging extraction from natural sources was highly desirable. Thus, Carreira and coworkers reported in 2019 the first gold-catalyzed asymmetric total synthesis of (−)-Merochlorin A **151** [113]. Starting from propargylcarboxylate **152**, the bicycle[3.3.0]octane derivative **153** was obtained via an enantiospecific gold-catalyzed tandem 1,3-acetoxy transfer followed by Nazarov cyclization and aldol reaction in a single step (Scheme 36). [JohnPhosAu(CH_3_CN)]SbF_6_ provided the best results, affording compound **153** in 88% after an additional hydrolysis step. Finally, seven additional steps permitted the obtention of (−)-Merochlorine A **151**.

**Scheme 37:**
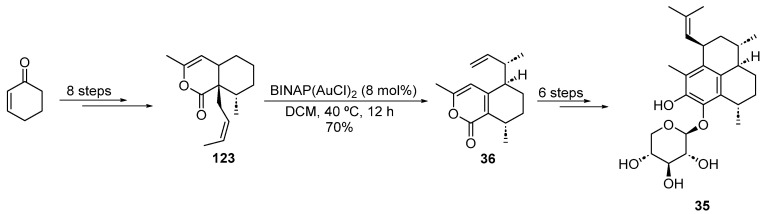
Gold-catalyzed Cope rearrangement in the total synthesis of Pseudopterosin A **35**.

As stated before, Pseudopterosins are promising drugs for their anti-inflammatory and analgesic effects [58]. In the previously described synthesis of Pseudopterosin A **35** (see Scheme 29), two key gold-catalyzed steps were identified, consisting of the first one in an alkyne hydrocarboxylation and the second one in a Cope rearrangement. Compound **123**, previously obtained via gold catalysis, can rearrange to furnish 5,6,7,8-tetrahydro-1*H*-isochromen-1-one **36** in 70% yield in the presence of 8 mol% of a 1:1 mixture of BINAP(AuCl)_2_/AgNTf_2_ in DCM at 40 °C (Scheme 37). Finally, six additional steps allowed them to obtain the desired product Pseudopterosin A **35**.

**Scheme 38:**
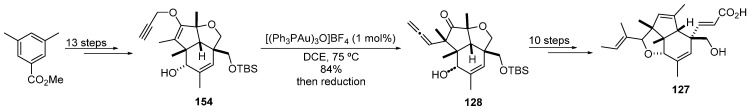
Gold-catalyzed Claisen rearrangement in the total synthesis of Indoxamycin B **127**.

The previously commented total synthesis of Indoxamycin B **127** (Scheme 31) developed by Carreira and coworkers [101] comprised two key gold-catalyzed steps: a propargyl Claisen rearrangement and an allene hydroalkoxylation. The 1,5-enyne **154** necessary for the gold stage could be obtained in 12 synthetic steps. After a careful screening, it was found that a 1 mol% [(Ph_3_PAu)_3_O]BF_4_ in DCE at 75 °C afforded the best result, furnishing compound **128** in 84% after in situ reduction (Scheme 38). Then, the previously explained gold-catalyzed hydroalkoxylation (see Scheme 31) and another nine additional steps generated the final product Indoxamycin B **127**.

#### 2.3.4. Gold-Catalyzed Glycosylation

Given the extremely diverse biological activities of naturally occurring glycans, a wide array of glycosylation methods has been developed to access novel artificial analogs with enhanced properties. These methods mainly involve the substitution of a leaving group on the anomeric carbon of a glycosyl donor with a nucleophile under the action of usually a stoichiometric electrophile. However, glycosylations are still problematic since some species that exist in the leaving group and the promoter can interfere. In this regard, gold(I) catalysts allowed to overcome these issues not long ago [114]. Glycosyl *o*-alkynylbenzoates turned out to be privileged donors under gold(I) catalysis with Ph_3_PAuNTf_2_ and Ph_3_PAuOTf due to their easy preparation and stability, wide substrate scope, lower undesired reactivity, simplicity and orthogonality to conventional methods.

**Scheme 39:**
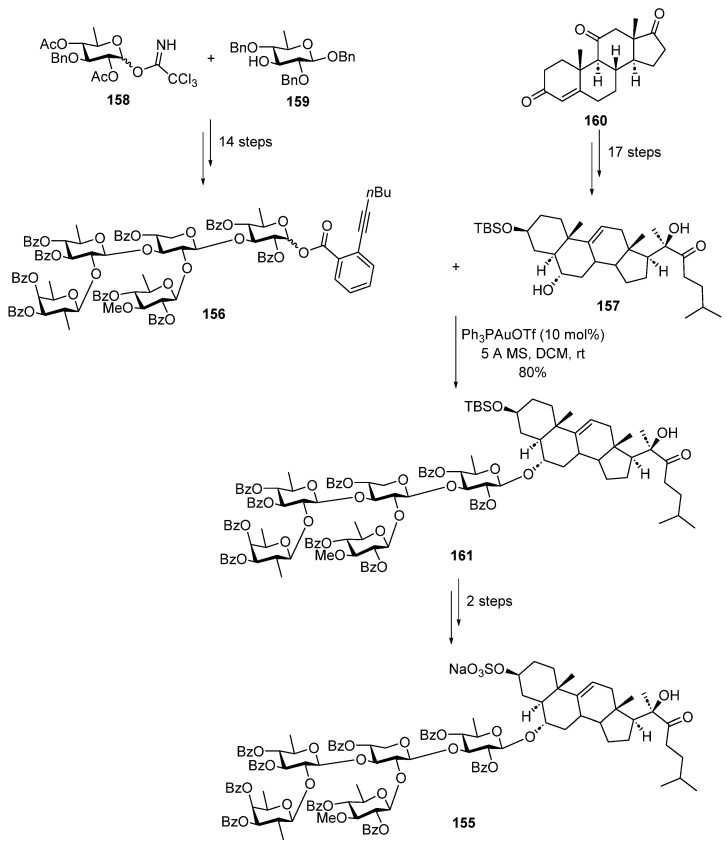
Gold-catalyzed glycosylation in the total synthesis of Goniopectenoside B **155**.

A good example of these advantages was reported in the total synthesis of Goniopectenoside B **155** reported by Yu and coworkers [115]. Isolated from *Goniopecten demonstrans* starfish, this steroid glycoside displayed interesting antifouling activity [116], although more types of activities are expected. The precursors for the gold-catalyzed step, pentasaccharide **156** and steroid **157**, can be prepared from **158**, **159** and **160** in 14 and 17 steps, respectively. The use of 20 mol% Ph_3_PAuOTf and 5 Å molecular sieves in DCM at room temperature afforded product **161** in 80% yield. Two additional steps completed the total synthesis of Goniopectenoside B **155**, becoming the first synthesized marine steroidal glycoside, with an overall 4.3% yield (Scheme 39).

### 2.4. Other Transition-Metal-Catalyzed Transformations in the Total Synthesis of Marine Drugs

#### 2.4.1. Scandium Catalysis

The application of scandium complexes in organic chemistry was historically limited until the introduction of Sc(OTf)_3_ as Lewis acid [117]. Since then, the unique characteristics of Sc(III) triflate complexes, including stability, recovery and reusability, strong Lewis acidity and environmental benignity, have fostered continuous attention and fruitful research in scandium-catalyzed processes [118]. In this regard, scandium-catalyzed domino reactions, Michael additions, Mannich reactions, cycloaddition reactions, ring-opening of epoxides and other transformations have been reported [119]. Over the past two decades, scandium asymmetric chemistry has become crucial in asymmetric synthesis, showcasing the versatility and impact of this early transition metal in total synthesis [119].

**Scheme 40:**
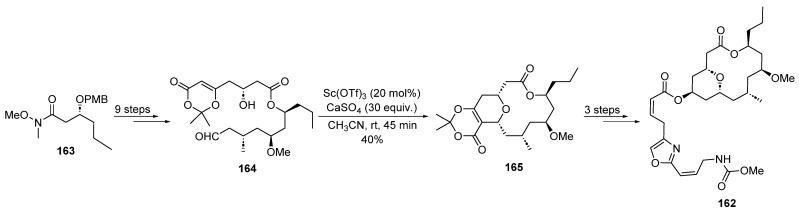
Sc-catalyzed macrocyclization in the total synthesis of (+)-Neopeltolide **162**.

Regarding marine-derived natural products, an interesting example of a Sc-catalyzed macrocylization has been employed by Scheidt and coworkers for the total synthesis of (+)-Neopeltolide **162** [120]. This compound, a marine-derived macrolide isolated from deep-water *Neopeltidae* sponges [121], is a highly cytotoxic and potent inhibitor of tumor cell proliferation in vitro, a cytostatic dose-dependent agent and a powerful antifungal [121]. Starting from amide **163**, the authors were able to obtain the compound **164** necessary for the key Sc-catalyzed macrocyclization step in nine linear steps. The use of 20 mol% Sc(OTf)_3_ in acetonitrile at room temperature afforded the macrolide core fragment **165** in 40% yield (Scheme 40). As noted by the authors, the remarkable degree of generated complexity compensates for the modest yield. Three additional steps were necessary to achieve the natural product (+)-Neopeltolide **162**.

#### 2.4.2. Titanium Catalysis

Titanium, as the second most abundant transition metal on Earth, holds promising significance in inexpensive metal catalysis [122]. Compared to late transition metals like nickel, its by-products such as TiO_2_ are generally considered harmless and non-toxic [123]. Titanium’s catalytic prowess is evident in olefin polymerization, crucial for industrial polyethylene production [124]. Its complexes, though highly reactive in diverse bond activation reactions, can face limitations due to oxophilic character and moisture sensitivity. Nonetheless, titanium’s low toxicity and biocompatibility [125] make it attractive for fine chemical synthesis, prompting recent advancements in its catalyst development [126].

**Scheme 41:**
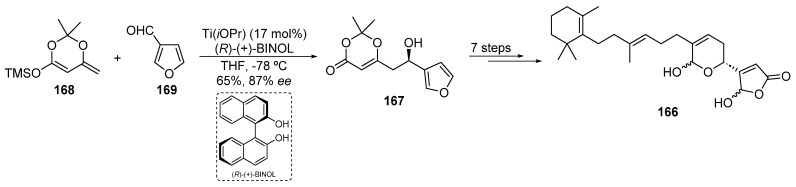
Ti-catalyzed aldol condensation reaction in the total synthesis of Manoalide **166**.

Since the pioneering work by Mukaiyama and coworkers [127], titanium species have been widely employed as Lewis acid catalysts, particularly for aldol condensation reactions. This fact led to consecutive improvements in the area, culminating in enantioselective transformations employing chiral ligands for the Ti center [128]. Taking advantage of this reactivity, one step in the total synthesis of the marine natural product Manoalide **166** has been achieved. Manoalide, a non-steroidal ester extracted from sponge *Luffariella variabilis*, has shown interesting activity as a phospholipase A2 inhibitor and anti-inflammatory, anti-cancer and anti-virus activity [129,130,131,132]. Its main structural feature consists of a six-membered hemiacetal ring and a γ-lactone ring. The first enantioselective total synthesis of Manoalide **166** was reported by Scettri and coworkers [133] employing Ti catalysis for the construction of a key fragment in the synthesis. The Ti-catalyzed step takes place early in the route, affording the coupled product **167** from **168** and **169** in 65% yield with high enantioselectivity (Scheme 41). Seven additional steps completed the total synthesis of compound **166**.

In recent years, titanium has emerged as a versatile catalyst for organic synthesis through formal redox transformations, enabling various C–C, C–O and C–N bond formations [134,135], and hydrofunctionalization catalysis, focusing on hydroamination and hydroaminoalkylation of C–C multiple bonds [136]. These reactions are utilized for constructing selectively substituted amines and *N*-heterocycles, commonly found in biologically active compounds such as natural alkaloids, pharmaceuticals, and agrochemicals [137].

**Scheme 42:**
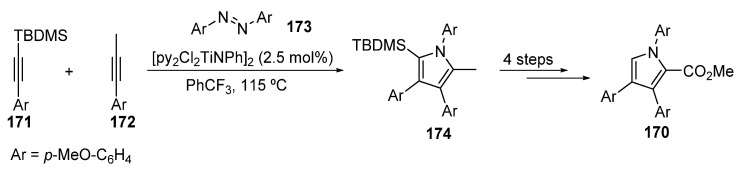
Ti-catalyzed [2+2+1] pyrrole synthesis used for accessing Lamellarin R **170**.

A recent example reported by Tonks and coworkers showcased the ability of Ti-catalyzed [2+2+1] pyrrole synthesis to access the marine natural product Lamellarin R **170** [138]. Compound **170**, firstly isolated from *Dendrilla cactos* sponge [139], belongs to the Lamellarins family, a group of marine alkaloids with a densely functionalized pyrrole core that exerts a wide range of biological activities: cytotoxicity against tumor cells, anti-HIV, multidrug resistance reversal activity, immunomodulatory, etc. [140]. In this approach, a silylalkyne **171** and a methyl-aryl alkyne **172** act as coupling partners in the presence of 2.5 mol% [py_2_Cl_2_TiNPh]_2_ as a catalyst and a suitable azoarene **173** as N-Ar source (Scheme 42). **174** was immediately submitted to four additional steps due to its instability, intersecting with a previously reported methodology [138,141] Lamellarin R **170**.

#### 2.4.3. Iron Catalysis

Iron holds a promising position in the future of catalysis given its high natural abundance in the Earth’s crust and the low toxicity of its salts [125]. Although initially associated with the Friedel–Crafts reaction, Fe complexes have found increasing application as catalysts, proving intriguing reactivity over decades beyond mere Lewis acid catalysis. Indeed, iron has shown its potential in cross-coupling, carbocylization and C-H bond activation reactions [142]. All these features would possibly make Fe species the future catalysts of choice for introducing C-C bonds in the synthesis of complex organic molecules, including natural products [143].

**Scheme 43:**
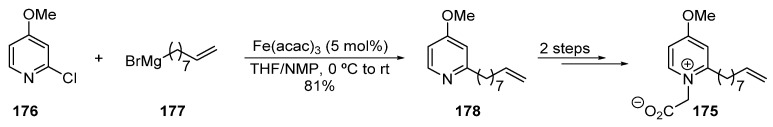
Fe-catalyzed cross-coupling reaction in the total synthesis of Montipyridine **175**.

One of the most employed Fe-catalyzed cross-coupling reactions involves the use of Grignard reagents and C_sp2_ electrophiles. Although this reaction was reported by Tamura and Kochi [144], it remained shadowed by the Pd-catalyzed Kumada–Corriu process [145,146]. However, an enormous advance has taken place in the field since the 2000s, and there can be found some examples of Fe-catalyzed cross-coupling in the total synthesis of marine natural products such as Montipyridine **175**. This pyridinium alkaloid isolated from stony coral *Montipora* species has exhibited cytotoxic effects [147]. The total synthesis of compound **175** was reported in 2002 by Fürstner and coworkers to illustrate the potential of this Fe-catalyzed transformation [148]. In the presence of 5 mol% Fe(acac)_3_, pyridine **176** and Grignard reagents **177** can be efficiently coupled in THF/NMP to yield product **178** with 81% yield (Scheme 43). Finally, two steps carried out in a one-pot manner afforded Montipyridine **175** in 74% overall yield.

It is important to remark that there are other examples of marine-derived natural products including at least one Fe-catalyzed cross-coupling step in their total synthesis. However, since they have not shown biological activity so far, no further discussion has been carried out.

#### 2.4.4. Cobalt Catalysis

Cobalt, the lightest element among group 9, was explored in cross-coupling catalysis in early works during the 1940s [149]. However, it has been systematically belittled in catalysis over the years compared with its heavier neighbors rhodium and iridium. This is quite surprising given the huge availability and biorelevance of Co species [125]. Nevertheless, this trend started to turn over the last decades, becoming a promising alternative towards more sustainable metal-catalyzed processes. In this regard, numerous novel Co-catalyzed transformations have been reported to date, including cross-coupling reactions, hydrofunctionalization processes of alkynes and alkenes, (carbonylative) cycloisomerizations, cycloadditions, dehydrogenative functionalizations, radical transformations and C-H bond functionalization, among others [150,151,152,153,154,155].

Despite no reports of Co-catalyzed processes in the synthesis of pharmaceuticals being found until the 2010s, their application in the synthesis of natural products has experienced significant growth in recent years [156]. In the area of natural products from natural sources, a couple of Co-catalyzed steps have been reported over the last years. As some of the target molecules did not present any kind of bioactivity so far, these examples have not been further discussed in the following paragraphs.

**Scheme 44:**
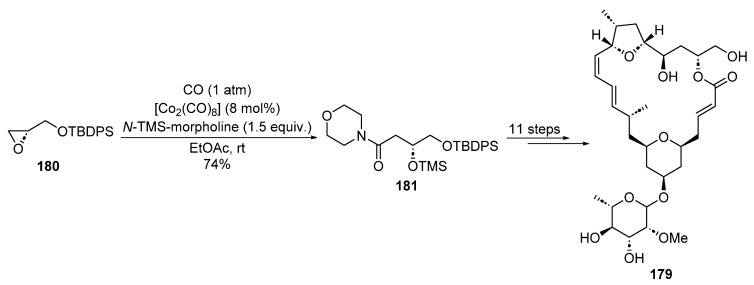
Co-catalyzed carbonylative ring opening of an epoxide in the total synthesis of Mandelalide A **179**.

Mandelalide A **179**, a marine macrolide isolated from *Leiodermatium* sponge, has shown cytotoxic activity against some cancer cell lines [157,158]. Fürstner and coworkers reported in 2014 a total synthesis of this marine natural product [159], involving a key Co-catalyzed step. In the presence of 8 mol% of Co_2_(CO)_8_ in EtOAc at room temperature, chiral epoxide **180** experienced a carbonylative ring-opening reaction with *N*-TMS-morpholine to furnish product **181** in 74% yield (Scheme 44). The subsequent 11 steps gave Mandelalide A **179** with a 4.5 % total yield.

**Scheme 45:**
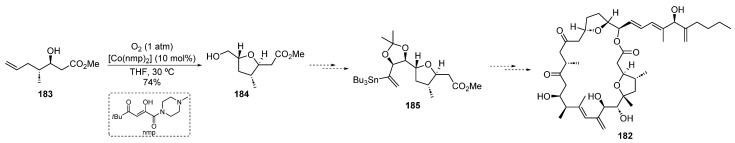
Co-catalyzed oxidative cyclization step in a hypothetic total synthesis of Amphidinolide C **182**.

Amphidinolides, isolated from the symbiotic marine dinoflagellate *Amphidinium* sp., are a family of macrolides containing linear polyketides [160]. Due to the macrocyclic structure, they possess interesting biological activity. For example, Amphidinolide C **182** shows potent biological activities against murine lymphoma and human epidermoid carcinoma cells [161]. Pagenkopf and coworkers carried out the total synthesis of a precursor for Amphidinolide C **182** in 2013 employing a Co-catalyzed aerobic oxidative cyclization step [162]. In the aforementioned reaction, compound **183** is cyclized in the presence of 10 mol% Co(nmp)_2_ and O_2_ to provide tetrahydrofuryl alcohol **184** in 74% yield (Scheme 45). Although the authors did not complete the synthetic sequence, compound **184** could afford the desired natural product **182** after some additional steps based on similar approaches for other amphidinolides, involving intermediate **185** [163].

#### 2.4.5. Nickel Catalysis

As noted by the Nobel Laureate Paul Sabatier in 1922, nickel remains the “spirited horse” among transition metals, due to both the exceptional catalytic power and the challenge of controlling nickel catalysts effectively [164]. The high reactivity of Ni species, the facile homolytic cleavage of the Ni-C bond and the readily accessible oxidation states from 0 to +3 give Ni species the ability to perform transformations beyond other transition metals scope with high efficiency, activating all kinds of strong bonds. On the contrary, the same features result in difficulty in controlling and predicting the outcome of Ni-catalyzed processes, along with an easy poisoning of the catalyst [164]. Over the years, fine-tuning of ligands has been crucial in designing homogeneous, more robust nickel catalysts [165,166,167], making outstanding discoveries in the field.

For instance, Ni-catalyzed cross-coupling reactions have revolutionized organic synthesis since the 1980s [168]. The careful choice and design of ligands have allowed the description of Ni-catalyzed analogs of the “traditional” Pd-catalyzed coupling, such as Kumada–Corriu, Suzuki–Miyaura, Negishi and Hiyama [169,170,171,172]. Other types of typically Pd-catalyzed C–X cross-coupling [173,174] and Mizoroki–Heck reactions [175,176] have been also unlocked for Ni species. Additionally, nickel catalysts have excelled in cyclizations, cycloaddition reactions and multicomponent couplings by activating unsaturated organic molecules [164]. Their ability to coordinate unsaturated molecules activates them towards unusual reactivity.

All these reasons have prompted the use of Ni catalysts in total synthesis of natural products [8,177,178]. In the field of marine-derived natural products total syntheses, a few examples of Ni-catalyzed steps can be found. As some of the target molecules did not exhibit interesting biological activity so far, they have not been discussed in the following paragraphs.

**Scheme 46:**
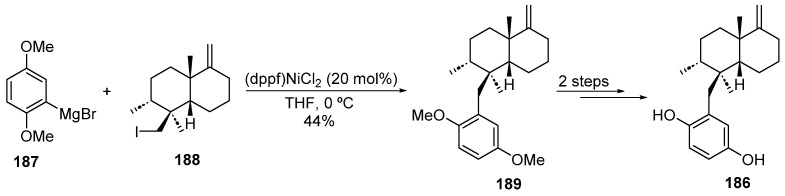
Ni-catalyzed cross-coupling step in the total synthesis of Arenarol **186**.

Arenarol **186** is a sesquiterpenoid compound isolated from the marine sponge *Dysidea arenaria* [179]. This marine compound has shown promising activity against human melanoma cells [179], making it an interesting target for the discovery of new drugs. Wiemer, Scott and coworkers were able to perform the total synthesis of this molecule in 1995 by means of Negishi-type Ni catalysis [180]. Starting from Grignard reagent **187** and iododecaline derivative **188**, product **189** was obtained in 44% yield using 20 mol% Ni(dppf)Cl_2_ in THF at 0 °C (Scheme 46). This remarkable transformation demonstrates the usefulness of Ni species in catalyzing C_sp3_–C_sp2_ cross-coupling for bulky substrates, usually a challenging transformation [181,182]. The target natural product Arenarol **186** was obtained after two more synthetic steps.

**Scheme 47:**
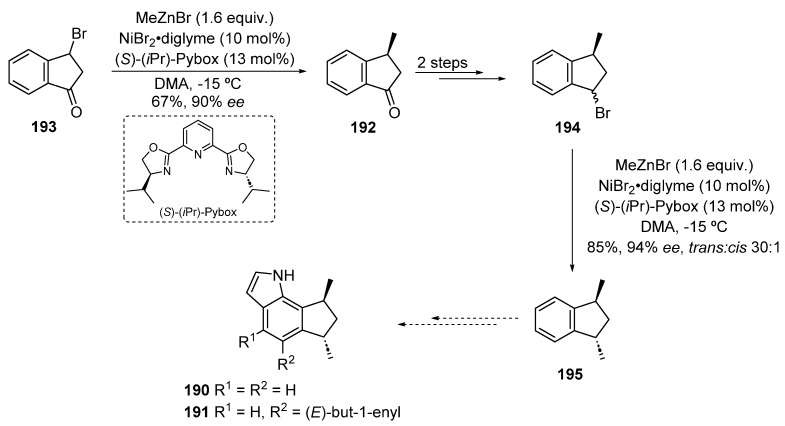
Ni-catalyzed Negishi coupling for the proposed enantioselective synthesis of marine natural products **190**–**191**.

The same methodology has been proposed as a selective alternative for the total synthesis of two marine-derived tetrahydrocyclopenta[*g*]indoles, *Trans*-trikentrin A **190** and Iso-*trans*-trikentrin B **191**. Both marine indole derivatives were isolated from *Trikentrion flabelliforme* sponge and exhibited antimicrobial activity [183]. The total synthesis of these derivatives carried out by MacLeod and coworkers employed a racemic substrate **192** [184,185], but Fu and Arp reported a Ni-catalyzed procedure to prepare precursor **192** in an enantioselective manner [186]. Starting from readily available bromoindanone **193**, an initial Ni-catalyzed Negishi coupling would afford product **192** in 67% yield with excellent enantioselectivity (>90% *ee*) (Scheme 47). Two more steps afforded bromoindane **194**, which was employed as starting material for the same Negishi coupling reaction to furnish compound **195** in 85% with excellent enantio and regioselectivity (94% *ee*, *trans:cis* 30:1). In both cases, MeZnBr was used as nucleophile, 10 mol% NiBr_2_-diglyme as catalyst, 13 mol% (*S*)-(*i*Pr)-Pybox as chiral ligand and DMA as solvent at −15 °C. Although the authors did not finish the total synthesis, they envisioned that the already reported route would afford *Trans*-trikentin A **190** and Iso-*trans*-trikentin B **191** in an enantioselective way.

Recent years have witnessed a renaissance in the development of new Ni-catalyzed reactions, leading to numerous applications in synthetic organic chemistry. This resurgence in nickel catalysis has revitalized old nickel salts and inspired new reactivity patterns by gaining insights into the nature of their mechanisms. Despite the vast literature on nickel catalysis, some fundamental aspects are still challenging, and promising directions will prompt their continued use in the quest for more sustainable processes.

#### 2.4.6. Copper Catalysis

Copper, due to its high abundance, low price and toxicity, would represent a good alternative to other transition metals in cross-coupling reactions [122,123]. Although the stoichiometric Ullman reaction was developed more than 120 years ago [187], the first Cu-catalyzed reaction of alkyl electrophiles with Grignard reagents appeared in the 1970s [188]. Since then, copper-catalyzed cross-coupling reactions experienced a renaissance during the 2000s prompted by the introduction of *N*-heterocyclic carbenes (NHCs) as ligands, developing novel methodologies with a strong impact in total synthesis [189]. In particular, Cu catalysis has been quite useful in the formation of C–X (X =O, N, S) [190,191,192] and, more recently, C–B, C–Si and C–F bonds [193]. Regarding the total synthesis of marine natural products, some examples can be found in the bibliography.

**Scheme 48:**
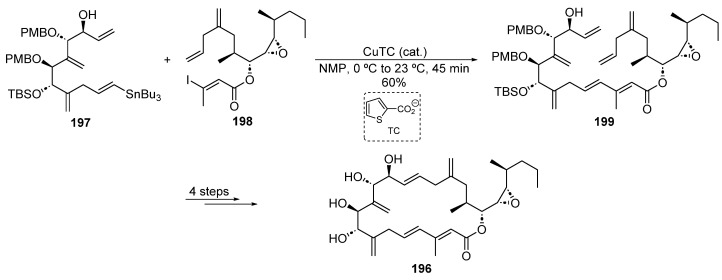
Cu-catalyzed cross-coupling reaction in the total synthesis of the proposed structure of Amphidinolide A **196**.

As stated before (see pp. 26 and 32), amphidinolides are a family of marine macrocylides with interesting biological properties. In this regard, Amphinolide A displays antineoplastic against leukemia [194]. Maleczka and coworkers performed a total synthesis of the proposed structure of Amphidinolide A **196** employing a Cu-catalyzed step [195]. After synthesizing fragments **197** and **198**, the cross-coupling reaction between them was found to happen faster and more efficiently under Cu catalysis rather than Pd catalysis. In fact, employing CuTC as a catalyst in NMP at 0 °C allowed authors to obtain the coupled product **199** in only 45 min in 60% yield (Scheme 48). After four additional steps, the presumed target molecule Amphidinolide A **196** could be obtained. However, the NMR data of compound **196** differed from that obtained for the isolated natural product.

#### 2.4.7. Zinc Catalysis

Zinc, the 24th most abundant element in the Earth’s crust, possesses non-toxic and environmentally benign properties [118,123], drawing attention during the last decades to their use as catalysts in organic chemistry [196]. In this regard, several types of Zn-catalyzed reactions have been reported to date, including aldol condensation, Henry reaction, Michael addition, alkynylations, hydrofunctionalizations of alkynes and cycloisomerizations, among others [196,197]. However, their predominant role in total synthesis remains as nucleophilic partners in cross-coupling reactions catalyzed by other transition metals. Nevertheless, Zn catalysis has been successfully employed in the total synthesis of some natural products and commercialized drugs [198,199]. In the field of marine natural products synthesis, some examples of Zn-catalyzed steps can be found in the bibliography, although they remain underexplored in comparison with other transition metals.

**Scheme 49:**
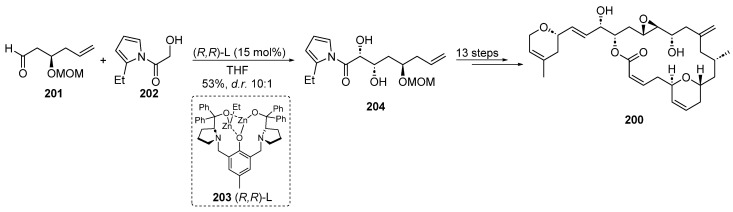
Zn-catalyzed aldol condensation in the total synthesis of Laulimalide **200**.

Laulimalide **200** is an intriguing marine macrolide with potent anticancer activity [200]. In their total synthesis approach, Trost and coworkers [201] pointed at a Zn-catalyzed direct asymmetric aldol-type reaction as one of the three key steps in this route (Scheme 49). Starting from readily available fragments **201** and **202**, the use of a carefully designed dinuclear Zn catalyst **203** allowed the formation of product **204** with excellent diastereoselectivity (>10:1 *dr*) and moderate yield (53% yield). Thirteen additional steps starting from **204** afforded the target natural product Laulimalide **200**.

#### 2.4.8. Molybdenum Catalysis

Molybdenum complexes can catalyze a wide range of organic reactions, as evidenced by the numerous reviews on the topic over decades [202,203]. In this regard, Mo complexes can perform different oxidation reactions (particularly alkenes epoxidation), nitrogen fixation, C-H bond activation and allylic alkylation. However, one of the reactions that showcases their efficiency and versatility is the metathesis reaction, particularly between alkynes [204]. However, Ru complexes are usually preferred in total synthesis for alkene metathesis, although some Mo-catalyzed examples can be found in the bibliography.

**Scheme 50:**
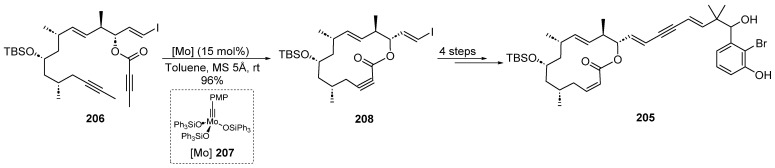
Mo-catalyzed RCAM step in the total synthesis of (+)-Callyspongiolide **205**.

In the field of marine natural products synthesis, most of the reported Mo-catalyzed steps comprise a ring-closing alkyne metathesis (RCAM) reaction followed by a selective hydrogenation to build large macrocyclic olefins. In this regard, Fürstner and coworkers have recently exemplified this chemistry through the total synthesis of (+)-Callyspongiolide **205** [205]. This natural macrolide isolated from a sponge of the *Callyspongia* genus holds promising activity against different human cancer cell lines [206]. After building compound **206** as a precursor, the RCAM can be performed using a 15 mol% Mo-carbyne complex **207** in toluene at room temperature, affording the macrocyclic compound **208** in an excellent 96% yield (Scheme 50). The total synthesis of (+)-Callyspongiolide **205** can be completed in only four additional steps after this transformation.

**Figure 5:**
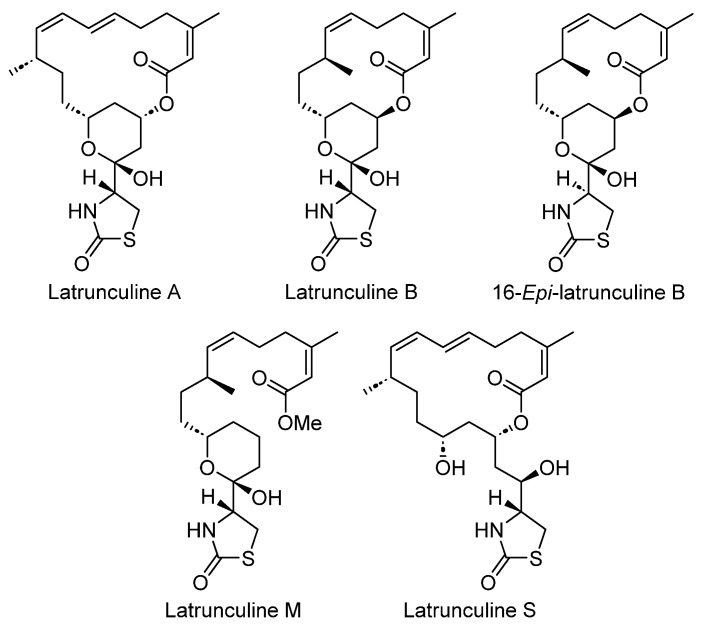
Molecular structure of Latrunculines synthesized via Mo-catalyzed RCAM.

Noteworthy, the same RCAM-hydrogenation strategy was previously employed for the total synthesis of other marine natural products. In this regard, several members of the macrolide Latrunculins family (Figure 5), isolated from the extract of the Red Sea sponge *Negombata magnifica* [207], have been synthesized following this procedure [208]. These natural compounds represent promising drugs due to their diverse effects, being particularly interesting for therapeutic application as anticancer agents [209].

**Scheme 51:**
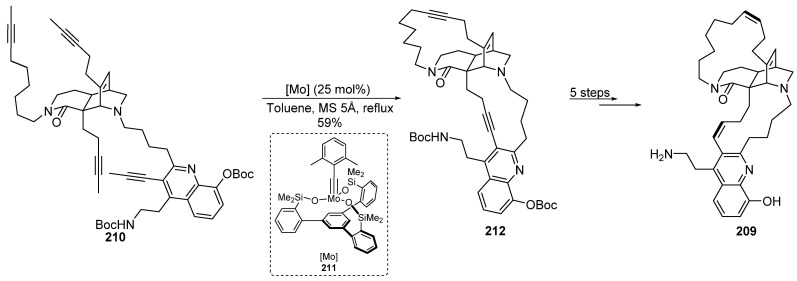
Mo-catalyzed RCAM step in the total synthesis of Njaoamine C **209**.

Remarkably, another example was reported in 2023 by Fürstner and coworkers [210] oriented to the total synthesis of Njaoamine C **209** (Scheme 51), a potent cytotoxic agent from the Manzamines family [211]. As an outstanding feature of this approach, a concomitant double RCAM takes place in the final stages of the synthesis, allowing to form simultaneously the two C-C bonds highlighted for Njaoamine C **209** in Scheme 51 from precursor **210**. The RCAM step takes place using a 25 mol% of the Mo carbyne complex **211** as a catalyst, affording macrocyclic intermediate **212** in a good 59% yield. Finally, five additional steps were required to complete the total synthesis of Njaoamine C **209**.

#### 2.4.9. Rhodium Catalysis

Rhodium, despite its scarcity and expensive price, has been one of the most extensively studied metals in catalytic transformation since the introduction of Wilkinson’s catalyst in 1967 [212]. The successful implementation of this complex to the catalytic hydrogenation of olefins spurred the development of other hydrofunctionalization reactions, area in which Rh complexes have shown outstanding performance [213,214,215]. In fact, Rh, Pd and Ir are the workhorses of the industry concerning hydrofunctionalization reactions [216].

Apart from hydrogenation and hydrofunctionalization processes, the general focus on Rh catalysis prompted the development of multiple novel transformations that showcased its ability to build C-C and C-X bonds while increasing molecular complexity. These transformations include allylation of alkynes and allenes, reductive C-C bond formation, cycloaddition reactions, cycloisomerizations, C-H bond functionalization, C-C bond activation, oxidation reactions and cyclopropanation, among others [215,217,218,219]. Given this long successful career, Rh catalysis has been widely implemented in the total synthesis of natural products. In this regard, some of the most employed transformations, as evidenced by numerous reviews of the topics, have been 1,4-addition to carbonyls [220,221], allylation reactions [222,223,224], C-H functionalization [225,226], cycloaddition and cycloisomerization processes [227]. Nevertheless, Rh-catalysis is still a hot area of research, and several Rh-catalyzed transformations are continuously increasing their applicability in total synthesis [228,229]. The total synthesis of marine-derived natural products has been also strongly impacted by the use of Rh catalysts, counting with numerous Rh-catalyzed steps. However, some of these target molecules have not shown potential therapeutic applications so far and, therefore, have not been disclosed in the next part.

**Scheme 52:**
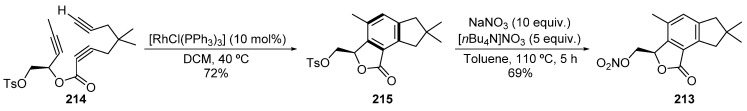
Rh-catalyzed cyclotrimerization step in the total synthesis of Alcyopterosin E **213**.

In an elegant approach, Witulski and coworkers were able to perform the first total synthesis of the marine sesquiterpenoid Alcyopterosin E **213** [230]. Alcyopterosins, isolated from deep-water Antarctic octocoral *Alcyonium* sp., have demonstrated mild cytotoxicity against some human tumor cell lines [231,232,233] and, in particular Alcyopterosin E, also anti-Leishmania activity [233]. The key step of this synthetic route relied on Rh-catalyzed alkyne cyclotrimerization to afford a benzene core with a challenging substitution pattern. In a late stage of the synthesis, compound **214** was treated with 10 mol% of Wilkinson’s catalyst [RhCl(PPh_3_)_3_] in DCM at 40 °C to furnish the cyclotrimerization product **215** in 72% yield (Scheme 52). One additional step would complete the synthesis to obtain Alcyopterosin E **213**, in a remarkably low number of steps.

**Scheme 53:**
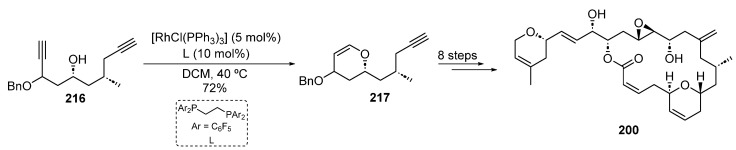
Rh-catalyzed cycloisomerization step in the total synthesis of Laulimalide **200**.

As stated before, Laulimalide **200** is an interesting marine natural macrolide with promising bioactivity. In the synthetic route previously disclosed (see Scheme 49), Trost and coworkers [234] devised a Ru-catalyzed alkene–alkyne RCM as a key step but also relied on a Rh-catalyzed cycloisomerization through hydroalkoxylation of an alkyne. Thus, starting from diyne compound **216,** the use of 5 mol% of [Rh(COD)Cl]_2_ and 10 mol% of the ligand in DMF afforded dihydropyran **217** in 55% yield (Scheme 53). Eight additional steps were required to convert **217** into the final product Laulimalide **200**.

#### 2.4.10. Iridium Catalysis

**Scheme 54:**
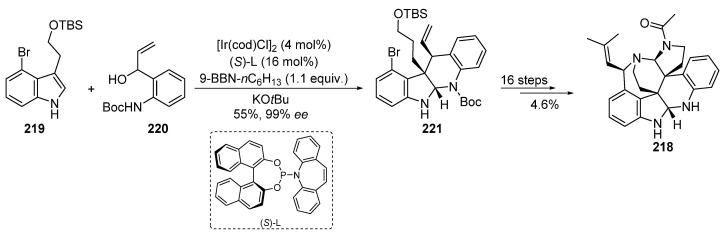
Ir-catalyzed cyclization step in the total synthesis of Communesin F **218**.

The journey of iridium complexes in catalysis has been quite sinuous since the beginning as reported by Crabtree [235]. In fact, the failure of the Ir analog of Wilkinson’s catalysts in performing catalytic hydrogenation led to an underestimation of this metal in comparison to Pd, Pt, Ru or Rh. Nevertheless, the implementation of different ligands designed to enhance the activity in Rh-catalyzed reactions enlightened the way for Ir species. The continuous study of Ir homogeneous catalysis deeply contributed to understanding the underlying mechanism behind crucial elemental organometallic steps such as oxidative addition [235]. The current scenario strongly differs from that several decades ago, as iridium plays a dominant role in organic synthesis catalyzing a myriad of different transformations, including asymmetric hydrogenation, hydrogen transfer reactions, C–H bond functionalization, asymmetric allylic substitution, cycloaddition reactions, enantioselective polycyclizations, photoredox catalysis and visible-light-induced catalysis, among others [235]. For these reasons, Ir complexes have found great application in the field of total synthesis [236].

(−)-Communesin F **218**, isolated from marine *Penicillium* fungi, is an indole alkaloid with significant cytotoxic and insecticidal activities [237]. This activity stems from the presence of a synthetically challenging polycyclic core bearing five different stereocenters. Its first enantioselective total synthesis was recently achieved by Yang and coworkers, relying on an Ir-catalyzed asymmetric intermolecular cascade cyclization [238]. As depicted in Scheme 54, fragments **219** and **220** could be cyclized in the presence of 4 mol% [Ir(cod)Cl]_2_ as a photocatalyst, (*S*)-L as ligand and 9-BBN-*n*C_6_H_13_ as an additive to generate product **221** in 55% yield with 99% *ee*. Notably, the authors were able to perform a gram-scale synthesis of compound **221** with no erosion of the yield or enantioselectivity. After this transformation, a total of 16 additional steps afforded (−)-Communesin F **218** with an overall yield of 4.6%.

**Scheme 55:**
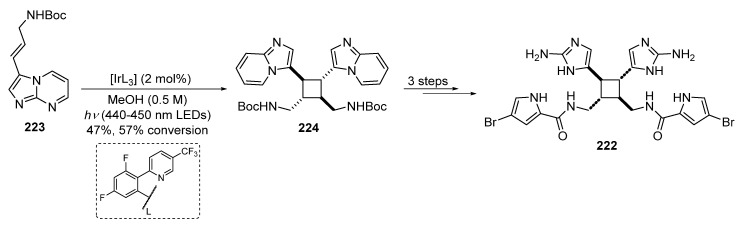
Ir-catalyzed VLI cyclodimerization in the total synthesis of (±)-Sceptrin **222**.

The marine natural product Sceptrin **222**, isolated from different genera of sponges, exhibited intriguing antitumor activity by inhibiting the motility of carcinogen cells [239]. In 2020, Nguyen and Jamison described a short synthesis of (±)-Sceptrin employing an Ir-catalyzed visible-light-induced (VLI) cyclodimerization reaction [240]. Under blue irradiation using 2 mol % of the iridium catalyst in methanol, compound **223** furnished cyclobutane dimer **224** in 41% yield with 57% conversion (Scheme 55). Remarkably, this process afforded one isomer selectively out of the 10 hypothetical possibilities, showcasing its key role in total synthesis. Four wisely designed steps converted compound **224** in the target molecule to (±)-Sceptrin **222** in high yield.

**Scheme 56:**
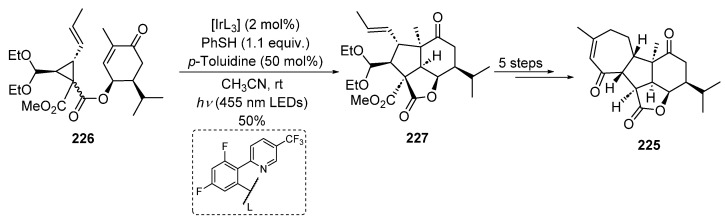
Ir-catalyzed VLI reaction in the total synthesis of (−)-Pavidolide B **225**.

Another good example of an Ir-catalyzed VLI process has been reported for the total of (−)-Pavidolide B **225**. This diterpenoid polycyclic natural product, isolated from the marine soft coral *Sinularia pavida*, represents an interesting scaffold since it exerts inhibitory activity against human leukemia cell line HL-60 [241]. A short enantioselective total synthesis of (−)-Pavidolide B **225** has been reported by Yang and co-workers employing Ir catalysis [242]. After synthesizing intermediate **226**, the key step takes place in the presence of 2 mol% of the catalyst, 50 mol% of *p*-toluidine and thiophenol as additive (Scheme 56). In this way, product **227** was isolated in 50% yield with high enantioselectivity. Noteworthy, preliminary studies employing Pd(0)-catalyzed cycloadditions were unsuccessful, demonstrating the importance of Ir catalysis in the overall process. Five additional steps completed the synthetic approach to (−)-Pavidolide B **225**, completing a concise 10-step enantioselective synthesis of this diterpenoid.

## 3. Conclusions

Transition metal complexes are strategically used in the creation of valuable precursors in multistep synthetic procedures for the preparation of marine drugs. These metal complexes provide good results in terms of yield and selectivity, in which multiple positions would be in principle susceptible to modification, but the metal is able to address the desired functionalization pathway. In addition, new connectivity patterns can be envisioned from the design of the synthetic route, which clearly facilitates the retrosynthetic analysis and allows to increase scaffold diversity. Of all the transition metals analyzed, palladium and ruthenium complexes are by far the most commonly used due to the powerful strategies already reported with these metals for the creation of C-C bonds, cross-coupling reactions and olefin metathesis, respectively. In addition, gold catalysis has provided exclusive carbo- and heterocyclization reactions for the construction of challenging scaffolds. We envisioned that the continuous development of new transition-metal-catalyzed methodologies combined with novel synthetic tools, such as photo- and electrochemistry, will be gradually incorporated into synthetic routes of marine drugs and their analogs to make the synthesis more accessible and extend the chemical space of their derivatives.

## Figures and Tables

**Table 1:** Most common transformations of olefin metathesis reaction in organic synthesis.

Reaction Type		Scheme
1	CM ^1^	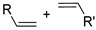
2	RCM ^2^	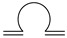
3	ROCM ^3^	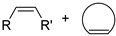
4	ADMEP ^4^	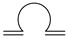
5	ROMP ^5^	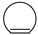
6	RCAM ^6^	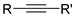

^1^ Cross Metathesis. ^2^ Ring Closing Metathesis. ^3^ Ring Opening Cross Metathesis. ^4^ Acyclic Diolefins Metathesis Polymerization. ^5^ Ring Opening Metathesis Polymerization. ^6^ Ring Closing Alkyne Metathesis.

## Abbreviations

acac: acetylacetonate; APIs: active pharmaceutical ingredients; aq.: aqueous; atm: atmosphere(s); 9-BBN: 9-borabicyclo(3.3.1)nonane; Bn: benzyl; Boc: *tert*-butyloxycarbonyl; Bpin: pinacol boronate; ^t^Bu: *tert*-butyl; *cf*.: *confer*/*conferatus;* COD: 1,5-biscyclooctadiene; dba: dibenzylidene acetone; DCE: 1,2-dichloroethane; DCM: dichloromethane; DMA: N,N-dimethylacetamide; DMF: dimethylformamide; DBU: 1,8-diazabicyclo(5.4.0)undec-7-ene; DMSO: dimethyl sulfoxide; dppf: 1,1′-bis(diphenylphosphino)ferrocene; d.r.: diastereoisomeric ratio; equiv.: equivalent(s); Et: ethyl; EtOAc: ethyl acetate; GI_50_: median growth inhibition; h: hour(s); HIV: human immunodeficiency virus; HPLC: high-performance liquid chromatography; HUVECs: human-umbilical-vein endothelial cells; hv: light irradiation; IC_50_: half maximal inhibitory concentration; LED: light-emitting diode; M: molar; Me: methyl; min: minute(s); MeOH: methanol; ml: milliliter; mol%: molar %; MOM: methoxymethyl ether; MRSA: methicillin-resistant *Staphylococcus aureus*; MS: molecular sieves; MS/MS: tandem mass spectrometry; Ms: mesyl/methanesulfonyl; NCI: National Cancer Institute; NMP: N-methyl pyrrolidinone; NMR: nuclear magnetic resonance; Ns: nosyl; NTf_2_: bis(trifluoromethanesulfonyl)imide; o-tol: 1,2-tolyl; pg: picograms; Ph: phenyl; *i*Pr: *iso*-propyl; PMB: *p*-methoxyphenyl; PMP: *p*-methoxyphenyl; rt: room temperature; SEM: trimethylsilylethoxymethyl; sp.: species; TBS/TBDMS: *tert*-butyldimethylsilyl; TEA: triethylamine; TES: triethylsilyl ether; Tf: trifluoromethanesulfonyl; TFA: trifluoroacetic acid; THF: tetrahydrofuran; TIPS: triisopropylsilyl ether; TM: transition metal; TMS: trimethylsilyl; Ts: tosyl; UV: ultraviolet; v-ATPases: vacuolar-type adenosine triphosphatase; VCAM-1: vascular cell adhesion molecule 1; VLI: visible light induced.
